# Exploring evidence of positive selection reveals genetic basis of meat quality traits in Berkshire pigs through whole genome sequencing

**DOI:** 10.1186/s12863-015-0265-1

**Published:** 2015-08-20

**Authors:** Hyeonsoo Jeong, Ki-Duk Song, Minseok Seo, Kelsey Caetano-Anollés, Jaemin Kim, Woori Kwak, Jae-don Oh, EuiSoo Kim, Dong Kee Jeong, Seoae Cho, Heebal Kim, Hak-Kyo Lee

**Affiliations:** Interdisciplinary Program in Bioinformatics, Seoul National University, Kwan-ak St. 599, Seoul, Kwan-ak Gu 151-741 Republic of Korea; Department of Animal Biotechnology, Chonbuk National University, Jeonju, 561-756 Republic of Korea; Department of Animal Sciences, University of Illinois, Urbana, IL 61801 USA; C&K genomics, Main Bldg. #514, SNU Research Park, Seoul, 151-919 Republic of Korea; Department of Animal Science, Iowa State University, Ames, IA 50011 USA; Department of Animal Biotechnology, Faculty of Biotechnology, Jeju National University, Ara-1 Dong, Jeju-Do, Jeju 690-756 Republic of Korea; Department of Agricultural Biotechnology, Seoul National University, Seoul, 151-742 South Korea

**Keywords:** Berkshire pigs, Selection signature, Meat quality, XP-EHH, XP-CLR, *de novo* assembly

## Abstract

**Background:**

Natural and artificial selection following domestication has led to the existence of more than a hundred pig breeds, as well as incredible variation in phenotypic traits. Berkshire pigs are regarded as having superior meat quality compared to other breeds. As the meat production industry seeks selective breeding approaches to improve profitable traits such as meat quality, information about genetic determinants of these traits is in high demand. However, most of the studies have been performed using trained sensory panel analysis without investigating the underlying genetic factors. Here we investigate the relationship between genomic composition and this phenotypic trait by scanning for signatures of positive selection in whole-genome sequencing data.

**Results:**

We generated genomes of 10 Berkshire pigs at a total of 100.6 coverage depth, using the Illumina Hiseq2000 platform. Along with the genomes of 11 Landrace and 13 Yorkshire pigs, we identified genomic variants of 18.9 million SNVs and 3.4 million Indels in the mapped regions. We identified several associated genes related to lipid metabolism, intramuscular fatty acid deposition, and muscle fiber type which attribute to pork quality (*TG*, *FABP1*, *AKIRIN2*, *GLP2R*, *TGFBR3*, *JPH3*, *ICAM2*, and *ERN1*) by applying between population statistical tests (XP-EHH and XP-CLR). A statistical enrichment test was also conducted to detect breed specific genetic variation. In addition, de novo short sequence read assembly strategy identified several candidate genes (*SLC25A14*, *IGF1*, *PI4KA*, *CACNA1A*) as also contributing to lipid metabolism.

**Conclusions:**

Results revealed several candidate genes involved in Berkshire meat quality; most of these genes are involved in lipid metabolism and intramuscular fat deposition. These results can provide a basis for future research on the genomic characteristics of Berkshire pigs.

**Electronic supplementary material:**

The online version of this article (doi:10.1186/s12863-015-0265-1) contains supplementary material, which is available to authorized users.

## Background

The domestic pig, *Sus scrofa domestica*, has been an important food source throughout human history. In addition to undergoing natural selection due to various environmental factors, pig breeds have gone through intensive artificial selection in order to increase economically important traits such as reproduction, growth rate, stress resistance, and meat quality [1]. For example, studies have shown that modern Landrace and Yorkshire breeds were positively selected to improve both reproduction and lactation ability for economic traits [2].

**Table 1:** Major candidate genes for meat quality detected from positive selection scans (XP-EHH and XP-CLR)

Candidate genes	Chromosome	Window (Mbp)	XP-EHH (B-L)^a^	XP-EHH (B-Y)^b^	XP-CLR (B-L)^c^	XP-CLR (B-Y)^d^
*TG*	4	8.075–8.1	6.62E-03	9.01E-03	316.91	512.22
*FABP1*	3	60.625–60.65	4.37E-03	8.11E-03	343.91	463.03
*ERN1*	12	14.95–14.975	4.71E-03	2.46E-03	334.99	358.46
*ICAM2*	12	14.95–14.975	4.71E-03	2.46E-03	334.99	358.46
*JPH3*	6	1.8–1.825	9.86E-03	7.81E-03	550.37	534.39
*TGFBR3*	4	136.675–136.7	4.64E-03	4.10E-03	167.01	230.76
*GLP2R*	12	57.45–57.475	8.35E-03	4.71E-04	177.41	277.77
*PPP2R5C*	7	129.925–129.95	6.45E-03	7.40E-03	179.71	184.98
*AKIRIN2*	1	62.775–62.8	6.07E-04	6.08E-03	138.15	120.63

^a^Empirical *P*-value resulting from XP-EHH analysis between Berkshire and Landrace

^b^Empirical *P*-value resulting from XP-EHH analysis between Berkshire and Yorkshire

^c^XP-CLR score of genomic region between Berkshire and Landrace

^d^XP-CLR score of genomic region between Berkshire and Yorkshire

**Table 2:** Predicted gene list related to meat quality from Berkshire specific aligned contigs

Predicted Ensembl ID	Gene symbol	contig length	Depth coverage^a^
*ENSSSCG00000012660*	*SLC25A14*	15,059	13.8
*ENSSSCG00000000857*	*IGF1*	8,107	17.7
*ENSSSCG00000006310*	*POU2F1*	19,766	11.9
*ENSSSCG00000010092*	*PI4KA*	29,825	17.0
*ENSSSCG00000017433*	*KRT14*	11,812	12.4
*ENSSSCG00000013754*	*CACNA1A*	45,820	11.0
*ENSSSCG00000015953*	*DLX1*	26,563	10.9
*ENSSSCG00000017589*	*DLX3*	26,563	10.9

^a^Average depth coverage of total mapped length in common between Berkshire samples

Berkshire pigs have been renowned for their superior meat quality since their meat contains a great proportion of neutral lipid fatty acids and marbling fat [3] which is important for palatability characteristics such as tenderness and juiciness. This breed has been intensively selected for meat quality in recent centuries, especially in East Asia where it is marketed as black pork at a premium price. Therefore, Berkshire has become specialized for high quality meat production and relative lack of boar taint following strong artificial selection for these traits. While several studies have investigated genetic factors relating to meat quality in Berkshire pigs [4–7], most of the research is performed in the traditional way using trained sensory panel analysis without investigating underlying genetic factors.

Recently, it has been shown that using a distorted pattern of genetic variation between populations can be useful for detecting selection related to specific traits. For example, genetic signals of selection discovered several genes in cattle responsible for milk production [8]. Also, Pollinger et al. identified rapid phenotypic diversification unique to the domestic dog [9], and Moradi et al. revealed three regions associated with fat deposition in thin and fat tail sheep breeds [10]. Thus, identifying genetic regions that are positively selected especially in Berkshire breed might allow us to reveal genetic variation related to phenotypic trait.

In this study, whole genome sequencing of Berkshire, Landrace, and Yorkshire breeds was conducted to identify genomic variants. We performed two statistical analyses, the cross-population extended haplotype homozygosity test (XP-EHH) and the cross-population composite likelihood ratio test (XP-CLR), to determine signals of selection in Berkshire breed. In addition, we performed a Fisher’s exact test for detection of breed specific amino acids or Indels, which are specifically enriched and affected by positive selection. Finally, Berkshire specific aligned reads were separately analyzed to detect the genomic difference between Berkshire and other breeds using *de novo* short sequencing reads assembly.

## Methods

### Ethics statement

The experiment and all its procedures were approved by the regional Ethical Committee (JNU Animal Bioethics committee permit number: 2013–0009).

### Sample preparation and whole genome re-sequencing

For genomic DNA extraction, tissue and blood samples were collected from 10 female Berkshire pigs. Berkshire tissue samples were collected from a local pig breeding company in Namwon, Korea. To generate inserts of ~300 bp, 3 μg of genomic DNA was randomly sheared using Covaris System. The TruSeq DNA Sample Prep. Kit (Illumina, San Diego, CA) was used for library construction by following the manufacturer’s guidelines. Whole genome sequencing was performed on the Illumina HiSeq 2000 platform. Whole-genome sequence data of 11 Landrace (Danish) and 13 Yorkshire (Large White) pigs was obtained from NCBI Sequence Read Archive database under accession number SRP047260. We used fastQC [11] software to perform a quality check on raw sequence data. Using Trimmomatic-0.32 [12], potential adapter sequences were removed prior to sequence alignment. Paired-end sequence reads were mapped to the pig reference genome (Sscrofa 10.2) from the Ensembl database using Bowtie2 [13] with default settings.

For downstream processing and variant-calling, we used open-source software packages: Picard tools (http://broadinstitute.github.io/picard/), SAMtools [14], and Genome Analysis Toolkit (GATK) [15]. “CreateSequenceDictionary” and “MarkDuplicates” Picard command-line tools were used to read reference FASTA sequence for writing bam file with only sequence dictionary, and to filter potential PCR duplicates, respectively. Using SAMtools, we created index files for the reference and bam files. We then performed local realignment of sequence reads to correct misalignment due to the presence of small insertion and deletion using GATK “RealignerTargetCreator” and “IndelRealigner” arguments. Also, base quality score recalibration was performed to get accurate quality scores and to correct the variation in quality with machine cycle and sequence context. For calling variants, GATK “UnifiedGenotyper” and “SelectVariants” arguments were used with the following filtering criteria. All variants with 1) a Phred-scaled quality score of less than 30; 2) read depth less than 5 ; 3) MQ0 (total count across all samples of mapping quality zero reads) > 4; or a 4) Phred-scaled *P*-value using Fisher’s exact test more than 200 were filtered out to reduce false positive calls due to strand bias.

We used “vcf-merge” tools of VCFtools [16] in order to merge all of the variants calling format files for the 34 samples. We used BEAGLE software [17] to conduct the haplotype phasing for the entire set of pig populations.

### Population stratification

We used Genome-Wide Complex Trait Analysis (GCTA) [18] to calculate eigenvectors which are equivalent to those estimated by the EIGENSTRAT software tool for principal component analysis (PCA). Autosomal genotype data was converted to PLINK [19] format, the input format required for GCTA, using VCFtools.

### Statistical analysis

Two methods were employed to infer positive signatures in Berkshire population. Firstly, XPEHH software [20], which measures cross-population extended haplotype homozygosity, was used to detect signatures of positive selection. We calculated EHH and the log ratio of the integrated haplotype homozygosity (iHH) for the pairwise test of Berkshire and other breeds for each of the SNP loci. An extreme value of XP-EHH suggests selection in Berkshire breed. We standardized log ratios using R [21], and divided the genome into consecutive, non-overlapping 25 kb windows. The SNP with the maximum XP-EHH value was selected to represent the summary statistics for each window. To define empirical *P*-value, we considered the number of SNPs in each window, and binned genomic windows according to the numbers of SNPs in increments of 200 SNPs. When a window encompassed more than 600 SNPs, we combined all the windows (>600 SNPs) into one bin. We defined an empirical *P*-value for each window based on its ranking of summary statistics in its bin following the protocol of previous studies [22, 23]. We assigned all of the regions with an empirical *P*-value less than 0.01 as the candidate regions which were positively selected in Berkshire breed.

Next, the cross-population composite likelihood ration test (XP-CLR) [24] was performed using the XP-CLR software package with non-overlapping windows of 25 kb. We designated windows with a XP-CLR value in the top 1 % of the empirical distribution as candidate regions. Genes located in the regions under significant selection were annotated. Additionally, we performed two types of Fisher’s exact tests using a 2x2 contingency table for detecting breed specific amino acid or Indel. Firstly, we performed a specific amino acid enrichment test using a contingency table composed of two factors such as specific breed (Berkshire/other ) and specific amino acid information (‘specific amino acid’/other). We performed the statistical test 3 (Berkshire, Landrace and Yorkshire) * k * n times on each of amino acid position in the targeted gene, where k is the number of existing different type of amino acid on each position, and n is number of site in targeted gene. Secondly, we performed a specific Indel enrichment test on the table composed of specific breed information and Indel existence (Yes/No) in each of positions on targeted gene. This statistical test was also performed 3*2*n times on each position. Using these tables, we performed a Fisher’s exact test with the alternative hypothesis that the odds ratio is greater than 1. The two types of statistical tests, for non-synonymous SNP and Indel, respectively, calculate cumulative type-1 error through individual statistical tests. The Bonferroni correction method was employed for considering multiple testing problems in the enrichment test.

### Short reads assembly using NGS sequence reads

To eliminate possible sequencing errors, we used “Error correction” module of Allpaths-LG [25] with default settings. Error corrected paired-end reads were merged to FASTA format using “Fq2fa” module from IDBA v1.1.1 software [26] which stands for iterative De Bruijn graph *De novo* assembler for short reads sequencing data with highly uneven sequencing depth. We assembled error corrected paired-end reads using IDBA_UD from IDBA package with the following parameters: 1) Perform pre-correction before assembly (“--pre_correction”), and 2) minimum k value should be more than 30 (−−mink 30). Using Gapcloser [27], we filled predicted gaps in the assembled sequences with a default setting.

In order to identify genomic regions unique to the Berkshire population, we defined sequence reads which unaligned to the reference genome and Landrace/Yorkshire assembled contigs but aligned to the Berkshire assembled contigs using Bowtie2 [13]. Among the total Berkshire assembled contigs, contigs with an average mapping depth of sequence reads resulted from the previous process of over 10 in common between every Berkshire samples were defined as the candidate region. RepeatMasker [28] was used to screen DNA sequences for interspersed repeats and low complexity DNA sequences before gene prediction for the candidate contigs.

## Results and discussion

### DNA sequencing and whole genome re-sequencing

The whole genomes of 10 Berkshire, 11 Landrace, and 13 Yorkshire pigs were sequenced to an approximate coverage of 11.68-fold on average, with a total of 1,201,160,368,944 bp in 11,981,734,530 reads after removing potential adapter sequence using Trimmomatic-0.32. Sequence reads of each breed were aligned to the pig reference genome (*Sus scrofa* 10.2) from the Ensembl database using Bowtie2, and 88.46 % of the sequence reads were aligned to the reference sequence (Additional file 1: Table S1–S3). After removing PCR duplicates and recalibrating base quality, 18,886,809 single nucleotide variants (SNVs) and 3,384,566 Indels were retained. Of the total SNVs, although 15,237,076 SNVs (80.7 %) have been already reported previously to dbSNP (Sus scrofa 10.2.74; ftp://ftp.ensembl.org/pub/release-74/variation/vcf/sus_scrofa/Sus_scrofa.vcf.gz), 3,649,733 SNVs were defined as novel variants (19.3 %). The distributions of both types of SNVs in each chromosome are shown in Additional file 2: Figure S1.

### Population stratification

**Fig. 1:**
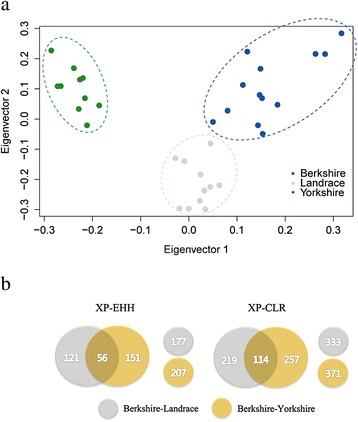
**a** Results of principal component analysis (PCA) of Berkshire, Landrace, and Yorkshire breeds. Eigenvector1 (x-axis) versus Eigenvector2 (y-axis). Both Eigenvector1 (15.7 % of the total variance) and Eigenvector2 (13.7 % of the total variance) indicate proportion of variance. **b** Summary of gene sets identified from statistical analyses (XP-EHH and XP-CLR) of Berkshire tested against Landrace and Yorkshire breeds

Using genome-wide complex trait analysis (GCTA), we performed principal component analysis (PCA) of the whole autosomal genotype loci (SNP; *n* = 18,802,810) to characterize the pattern of individual samples. The analysis revealed structurally cleared difference between populations. As shown in Fig. 1(a), the first eigenvector (15.7 % of the total variance) separated Berkshire from other breeds, and Landrace and Yorkshire pigs were divided by the second eigenvector (13.7 % of the total variance).

### Signatures of selection in the Berkshire breed

**Fig. 2:**
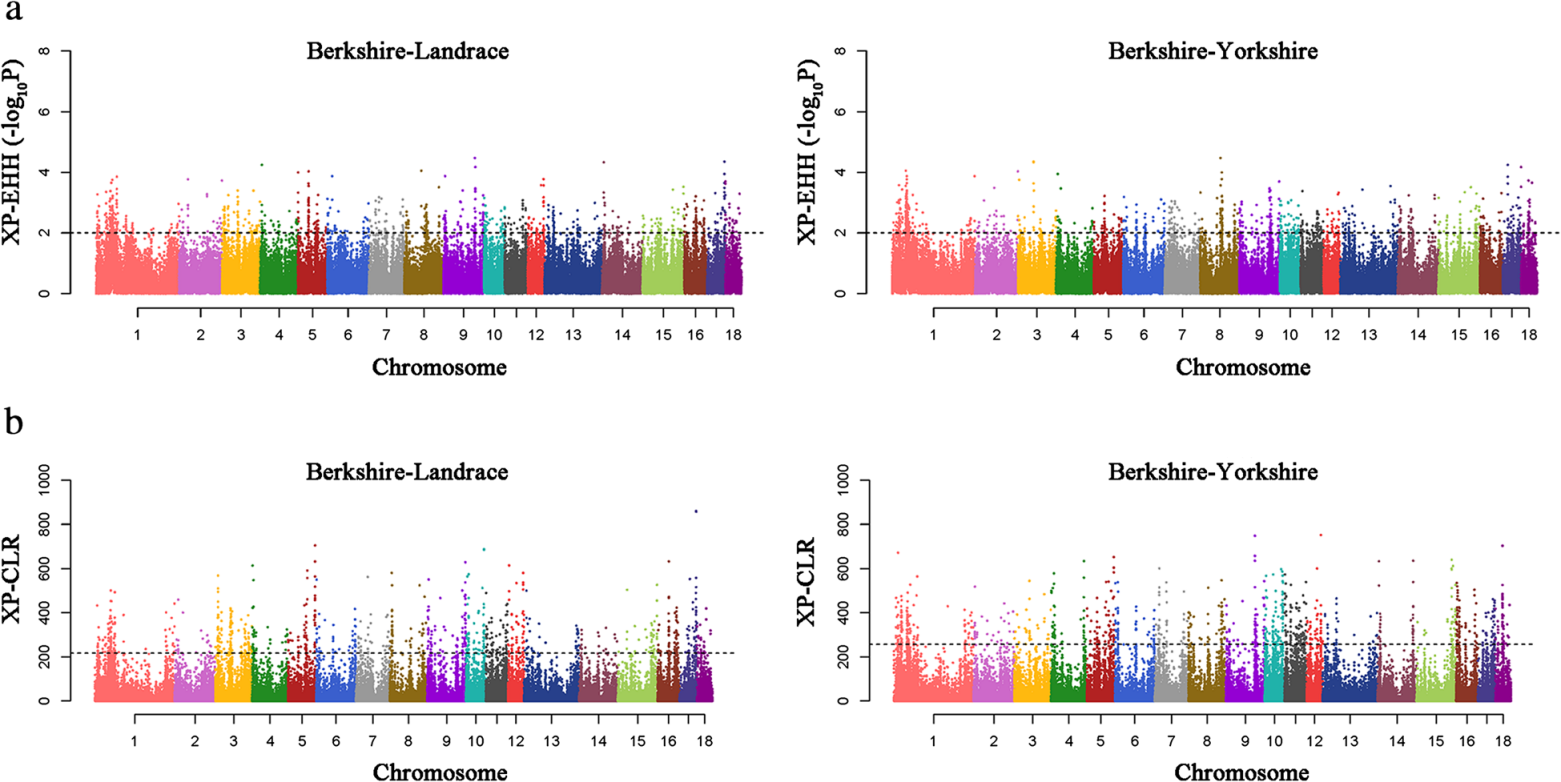
Results of two statistical analyses (XP-EHH and XP-CLR) are plotted across the genome. **a** Results of XP-EHH analyses and **b** Results of XP-CLR with Berkshire pigs against Landrace pigs or Yorkshire pigs for detection of positive selection signature. Each dot represents the maximum score in the non-overlapping 25 kb genomic region

To detect signals of positive selection in Berkshire against other breeds, we used two statistical analysis methods in order to achieve maximum statistical power for localizing the source of selection. We first used the cross-population extended haplotype homozygosity (XP-EHH) statistic to make comparisons between Berkshire and other breeds (Landrace and Yorkshire). This statistic is originally designed to estimate alleles that have increased in frequency to the point of fixation or near-fixation in one of the populations and assesses haplotype differences between two populations [29]. To make comparisons of genomic regions across populations, we divided the genome into consecutive, non-overlapping segments of 25 kb. Among the total of 98,037 windows, we assigned the maximum XP-EHH score in each segment as the window statistic. Giving consideration to the number of SNPs in each segment, the test statistic was converted to an empirical *p*-value based on its rank of XP-EHH score. Those that yielded significant values (*P* < 0.01) were identified as positively selected regions (Fig. 2(a)). A total of 177 and 207 genes were identified as positive signatures from XP-EHH test in Berkshire breed against to Landrace and Yorkshire breed, respectively (Fig. 1(b)).

We also ran a cross-population composite likelihood ratio test (XP-CLR) to search for the genomic regions where the changes in allele frequency at the locus occurred very fast due to random drift. XP-CLR is a multi-locus sliding window test which is robust to ascertainment bias in SNP discovery [24]. XP-EHH and XP-CLR were used to detect signatures of selective sweeps by comparing signals from two populations. However, while the XP-CLR test considers the variation of allele frequency using the differentiation of multi-locus allele frequency between two populations, the XP-EHH test aims primarily to identify differentially overrepresented haplotypes between two populations. In addition, combining the results from two different statistical analyses provides more powerful information than results from one test alone. We divided the whole genome area into non-overlapping windows of 25 kb as before. All windows above a threshold of 216.23 and 257.06 (top 1 % of the empirical distribution) were defined as significant regions (Fig. 2(b)), and identify 333 and 371 positively selected genes in Berkshire compared to Landrace, and to Yorkshire, respectively (Fig. 1(b)).

### Identification and analysis of positively selected genes in Berkshire

While selective traits are likely to be detected among various regions, we focused specifically on the meat quality specific to the Berkshire breed. The amount of fat and fatty acid in adipose tissue or muscle as well as the muscle fiber characteristic plays an important role in meat quality [3]. To identify genomic regions associated with meat quality in Berkshire, we detected candidate genes using two statistics (XP-EHH and XP-CLR) comparison between Berkshire and mother breeds (Landrace and Yorkshire) which are superior in maternal performance farrowing and raising large litters of pigs [30, 31]. Landrace and Yorkshire purebreds are well-known for their reproductive performance. In particular, Yorkshire pigs are noted for slow growth compared to Landrace or Berkshire pigs. When we compared the genes detected from statistical analyses of B-L and B-Y, a considerable number of common genes related to growth performance in the results of B-Y but not in the results of B-L (*WNT2, FGF14, PTPN11, FXYD2, APBB1, ACAP1, NET1, NF2,* and *KCTD11*).

We observed 56 genes (Additional file 1: Table S4) overlapped among the 177 and 207 resulting from comparisons between Berkshire and Landrace breeds and between Berkshire and Yorkshire breeds using XP-EHH analysis, respectively (Fig. 1(b)). The positively selected gene list included *FABP1* and *TG* (Additional file 1: Table S5). These results suggest that several genomic regions and genes may have been selected for meat quality in Berkshire pigs (Table 1). *Fatty acid-binding protein1* (*FABP1*) also known as liver fatty acid-binding protein (*L-FABP*) is a member of the *FABP* multi-gene family expressed in both the liver and small intestine [32]. It has been suggested that *L-FABP* gene, which has an effect on uptake, transport, mitochondrial oxidation, and esterification of fatty acids, were strongly related to meat quality in previous study [33–35]. *Thyroglobulin* (*TG*) gene, encoding to produce the precursor for thyroid hormones, affects adipocyte growth, differentiation and homeostasis of fat deports [36]. Many studies have shown that *TG* is significantly associated with meat quality traits. [37–40]. *AKIRIN2*, a homolog of the *Akirin* protein, is relevant to the control of skeletal myogenesis through up-regulation of muscle specific transcription factors [41]; it is also negatively regulated by cytokine such as myostatin, which plays an important role in skeletal myogenesis [42]. In a previous study, Sasaki et al. detected a SNP in the 3’ untranslated region of the *AKIRIN2* is associated with marbling in Japanese Black beef cattle [43]. The high proportion of marbling, which is defined by the amount and distribution of intramuscular fat (IMF), exceedingly improve the palatability by affecting the taste and tenderness of the meat. Also, a SNP located in an intron region of Glucagon-like peptide 2 receptor (*GLP2R*) is significantly associated with IMF according to a previous study [44]. Transforming growth factor β3 (*TGF-β3*), a secreted protein, is related with the mammalian target of rapamycin (mTOR) pathway, which has been renowned as significantly associated with muscle mass and strength [45]. Although its specific mechanism is not well understood, it is clear that *TGFBR3* plays a role in the muscular or adipose tissue development [46]. Also, Chen at el. recently discovered a SNP in *TGF-β1/2/3* had an effect on myofiber diameter [47]. Berkshire has been renowned to have smaller cross-sectional area and high density muscle fiber compared to other breeds [7]. Many studies have shown the relationship between the composition of myofiber type and pork quality [48], and this result is at the base of the fact that Berkshire pork has a tremendous tenderness and juiciness. Also, *JPH3*, *PPP2R5C*, *USP25*, and *ACTN2* were associated with boar taint [49], IMF, tenderness [50], and cooking loss [51], respectively.

To explore deep into the phenotypic traits of Berkshire breed, we further investigated the 114 genes (Additional file 1: Table S4) observed using XP-CLR (Fig. 1(b)). 13 genes intersected with the results from XP-EHH selection candidate genes (Additional file 1: Table S4). Interestingly, these genes included *FABP1*, *TG*, *ERN1*, *JPH3*, and *ICAM2* [52–55].

In addition to genes responsible for meat quality traits, our genome-wide selection scan also identified genes associated with immune response, particularly regulation of leukocyte and immunoglobulin (*CD79B*, *CD8B*, *FLT3*, *ICAM2*, *IFNGR1*, and *IGSF5*). Berkshire pigs have an unusually high concentration of plasma immunoglobulin as opposed to the other breeds, as evidenced by distinctive high percentages of neutrophils and leukocytes [56].

**Fig. 3:**
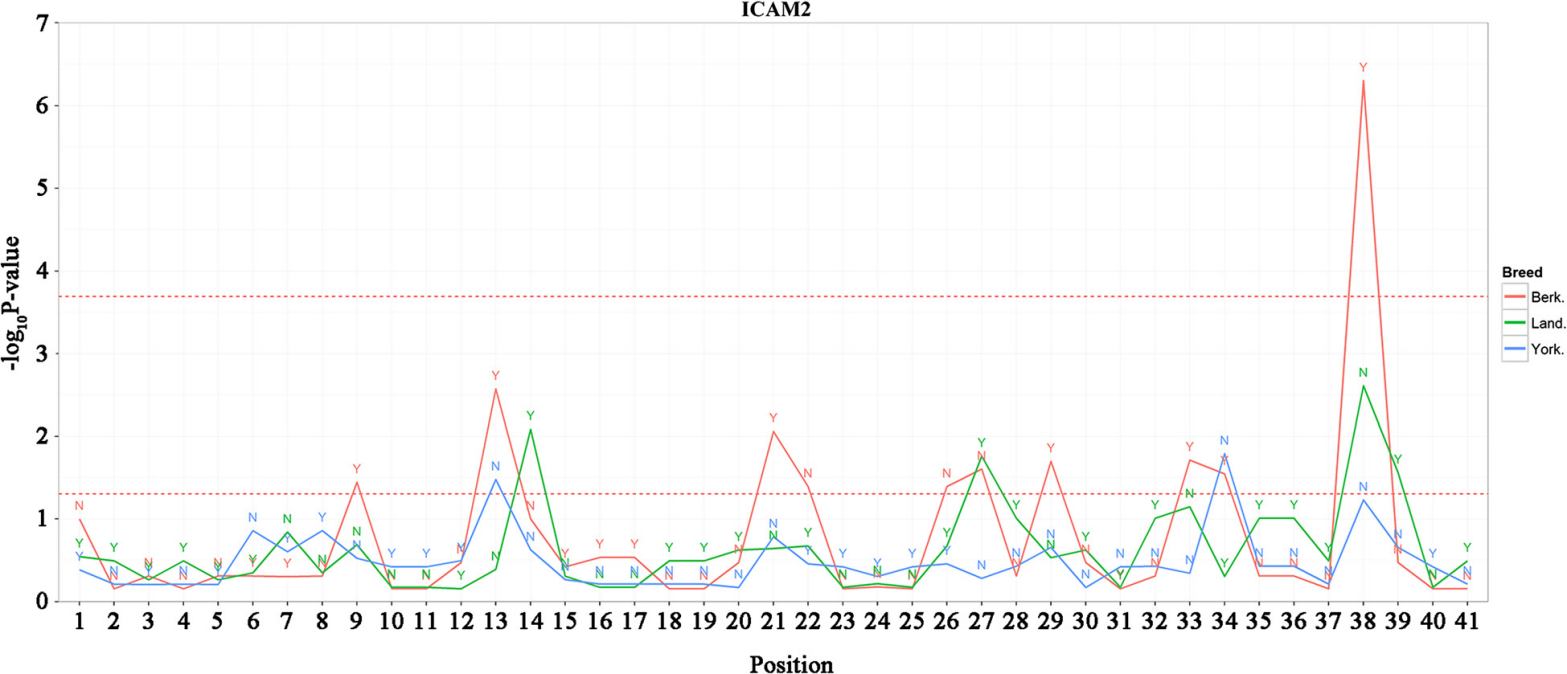
Results of Fisher’s exact test for detection of specific enriched sites on *ICAM2* gene (see Figure S2–S3 for other candidate genes). The X-axis represents the order of the detect variants in ICAM2 gene based on the reference genome. The Y-axis represents -*log*_10_ (*p-value*). The presence or absence of Indels is labelled as Y’s and N’s, respectively in each breed. The *upper red dotted-line* represents the Bonferroni cut-off line (5 % significance level) and the *lower red dotted-line* represents the 5 % significance level without multiple testing corrections

For further analysis of the influence of genomic variants on protein function, we performed a Fisher’s exact test for the detection of specific enriched sites on the 13 genes which were in the intersection with the results from XP-EHH and XP-CLR. Previously, most studies have focused on non-synonymous SNPs, since substitution is known to affect gene function. Also, many studies focused on deletions and insertions sites, which can affect the performance traits considerably in pigs [57, 58]. Therefore, we performed statistical analysis employing these two types of data, non-synonymous SNP and Indel site, under positive selective region. From the test results, numerous *P*-values are generated. For easily identified significant test results, we draw the line plots composed with -*log*_10_ (*p-value*) and each of site, y-axis and x-axis, respectively. Each test result was plotted together (Fig. 3; Additional file 2: Figure S2–S3). From the figures, we can easily detected significant enriched site, breed, and amino-acid or Indel, simultaneously. We identified several genes including significant sites, *TG*, *CPED1*, *CPNE8*, *CD8*, *ERN1*, *ICAM2*, *JPH3*, *NELFCD*, *SP110*, and *ADAM7* in Indel data under Bonferroni corrected 5 % significance level. These genes have a possibility that is related to breed specific phenotypic variation between Berkshire and other breeds by Indel.

### Whole genome assembly

Although analyzing positive selection signature between breeds using SNP and small Indel information could allow us to identify genetic variation which affects phenotypic diversity, it is also important to consider large sequence differences, which can be difficult to detect using reference-based alignments. We assembled short reads sequence of each breed to decipher the large genomic difference of Berkshire compare to other breeds more deeply. The sample with high concordantly paired mapping rate to the reference genome and with low heterozygosity was selected to perform genome assembly for each breed. After whole genome assembly was performed using IDBA-UD, all of the contigs less than 2,000 bp were removed for the minimum threshold length. We observed an average of 223,028 contigs with an average length of 10,843 bp, and N50 length for Berkshire, Landrace, and Yorkshire are 30,152, 9,379, and 9,694, respectively. We further performed the gapclosing step to fill N base within the contig. The average sum of the total assembled contigs after the gapclosing step for Berkshire, Landrace, and Yorkshire breeds was 2,304 Mbp, 1,927 Mbp, and 1,996 Mbp, respectively. Detailed results are shown in Additional file 1: Table S6.

To infer distinct genomic contents for Berkshire against other breeds, firstly, we compared the overall read mapping rate between assembled contigs for each breed, using the total mapped reads of each Berkshire sample (Additional file 2: Figure S4). The average overall read mapping rate to the Berkshire assembled contigs was 93.5 % in contrast to the Landrace and Yorkshire assembled contigs was 79.9 % and 82.3 %, respectively, which is also about 4.7 % higher to the overall mapping rate of reference-based alignment. Although satellites sequences were about 0.1 % in Berkshire assembled contigs which is about 0.04 % higher than others at 0.06 %, there was no significant difference based on the ratio of interspersed repeat elements including retrotransposon and retrovirus-like sequence in each assembled contigs (Additional file 1: Table S7).

We then separately remapped the each Berkshire sample’s sequencing reads, which were both unmapped to the reference genome and to the Landrace/Yorkshire assembled contigs, using Berkshire assembled contigs to find the regions in Berkshire that are distinct from the others. The average mapping rate of unmapped reads was about 37.8 % aligned to the Berkshire assembled contigs using Bowtie2, and the details of the information for each sample is described in Additional file 1: Table S8. Among the total number of 127,713 Berkshire assembled contigs, we observed 563 contigs which the unmapped reads were aligned with depth coverage of more than 10 in common between all Berkshire samples. Additionally, we removed PCR duplication of sequence reads to reduce the number false positives. As shown in Additional file 1: Table S7, the results summary of repeat contents demonstrated that high proportion of satellites (24.4 %) was detected in these contigs which is approximately 240 times higher than those of the total assembled sequence. After performing gene prediction and functional annotation, 43 contigs with 46 predicted genes were finally identified as Berkshire specific candidate genomic region. Out of 46 predicted genes, we identified 4 genes that were related to lipid metabolism: *SLC25A14* [59], *IGF1* [60], *PI4KA* [61], and *CACNA1A* [62] (Table 2). Li et al. recently identified 44 genes with 49 SNPs showing significant association with muscling and meat quality trait [51]. Of the 44 candidate genes, *DLX1* and *DLX3* showed a concordant result with our study. In addition, *TGFBR3* and *SYT1*, also identified from positive selection scan, were included in the candidate gene list. Besides the meat quality trait, 6 genes (*OR4D10*, *OR4D11*, *ENSSSCG00000028782*, *ENSSSCG00000029769*, *ENSSSCG00000013807*, and *ENSSSCG00000021192*) including 4 novel genes were related to olfactory receptor.

## Conclusions

Given the interest in the meat production industry of improving meat quality, genetic investigation of Berkshire pigs can provide information vital for selective breeding. Our analyses revealed several candidate genes that are involved in Berkshire meat quality including *TG*, *FABP1*, *AKIRIN2*, *GLP2R*, *TGFBR3*, *JPH3*, *ICAM2*, and *ERN1* from positive selection signature. Most of these genes are involved in lipid metabolism and intramuscular fat deposition. In addition, short sequence read assembly was conducted in order to investigate genetic variation which affects phenotypic diversity. Several candidate genes (*SLC25A14*, *IGF1*, *PI4KA*, and *CACNA1A*) were identified as contributing to lipid metabolism.

### Availability of supporting data

The sequencing data from this study has been archived at the NCBI Sequence Read Archive under BioProject [PRJNA: 281548].

## Additional files

Additional file 1: Table S1–S3. The result summary of sequence reads mapping using Bowtie2. **Table S4.** List of candidate genes resulted from genome-wide positive selection scan. **Table S5.** Information of genes which are previously reported as meat quality related genes. Descriptions of the gene functions are based on GeneCard. **Table S6.** The summary statistics of assembled contigs for Berkshire, Landrace, and Yorkshire using IDBA_UD. **Table S7.** The result summary of assembled contigs’ repeated and transposable elements for Berkshire, Landrace, and Yorkshire; and Berkshire assembled contigs of which unmapped reads were aligned. **Table S8.** The alignment mapping summary of unmapped sequencing reads to the Berkshire assembled contigs. (The unmapped sequencing reads were defined as the ones that were not mapped to the reference genome and to the Landrace and Yorkshire assembled contigs.). (DOCX 61 kb) Additional file 2: Figure S1. The distributions of novel SNV and known SNP in each chromosome. **Figure S2.** A statistical enrichment test (Fisher’s exact test) for detecting enriched non-synonymous SNP site on targeted genes. **Figure S3.** A statistical enrichment test (Fisher’s exact test) for detecting enriched Indel site on targeted genes. **Figure S4.** The overall reads mapping rate of assembled contigs for each breed and reference genome by aligning the total sequence reads of each Berkshire sample. (DOCX 3433 kb)

## Abbreviations

XP-EHH: The cross-population extended haplotype homozygosity test
XP-CLR: The cross-population composite likelihood ratio test
Indels: Insertions and deletions
GATK: Genome Analysis Toolkit
GCTA: Genome-Wide Complex Trait Analysis
PCA: Principal component analysis
IMF: Intramuscular fat
