# Supplementary material for: Exploring evidence of positive selection reveals genetic basis of meat quality traits in Berkshire pigs through whole genome sequencing
Source: BMC Genet. 2015 Aug 20;16:104. doi: 10.1186/s12863-015-0265-1 (PMC4545873; doi:10.1186/s12863-015-0265-1)

**Exploring evidence of positive selection reveals genetic basis of meat quality traits in Berkshire pigs through whole genome sequencing**

Authors: Hyeon Soo Jeong, Ki-Duk Song, Minseok Seo, Kelsey Caetano-Anollés, Jaemin Kim, Woori Kwak, Jae-don Oh, EuiSoo Kim, Dong Kee Jeong, Seoae Cho, Heebal Kim, Hak-Kyo Lee

| **ADDITIONAL FILE 1** |
| --- |

**Figure2-12**

Figure S12

Figure S23-6

Figure S37-11

Figure S412

**Figure S1**. The distributions of novel SNV and known SNP in each chromosome.


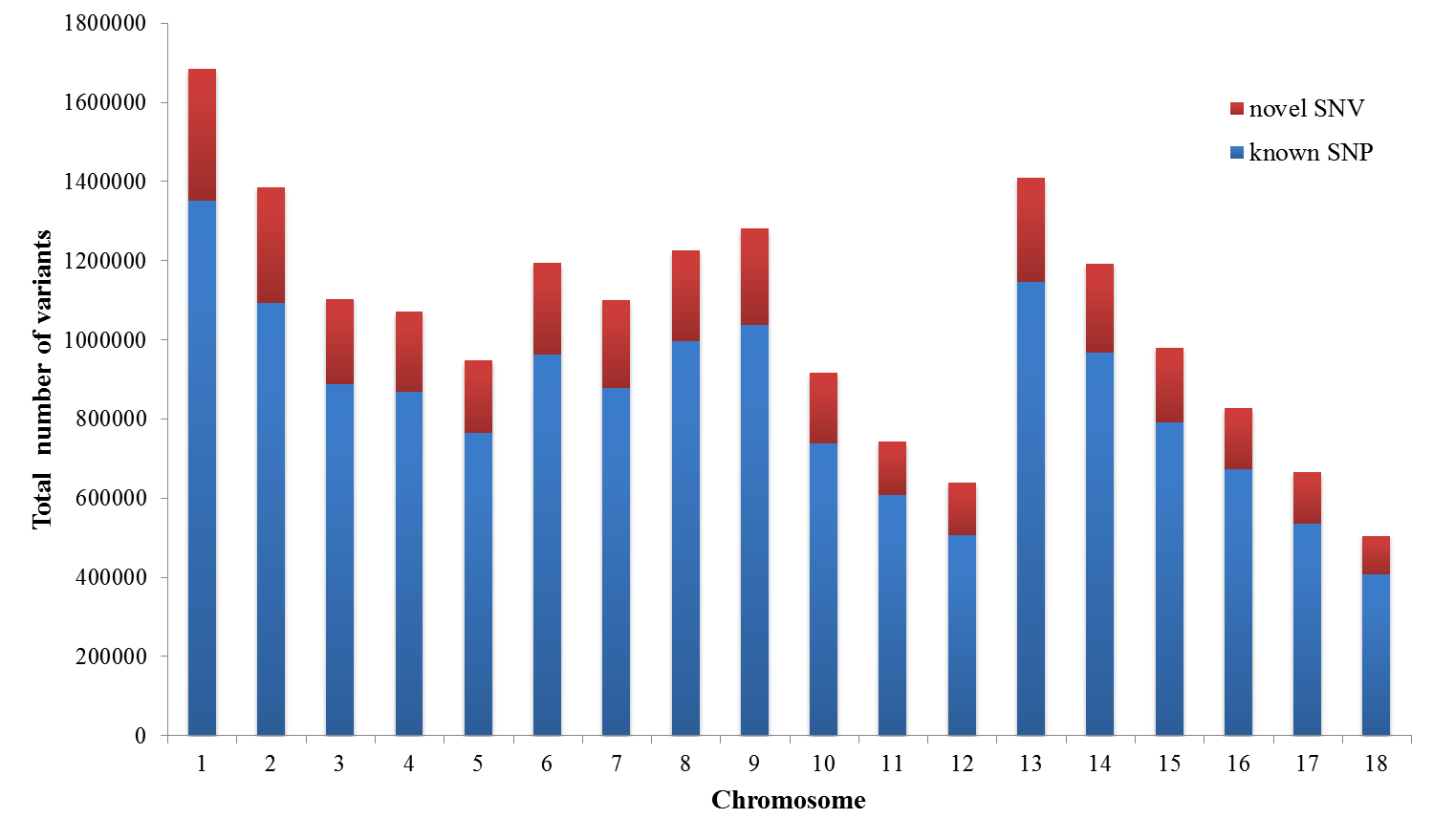


**Figure S2**. A statistical enrichment test (Fisher’s exact test) for detecting enriched non-synonymous SNP site on targeted genes.


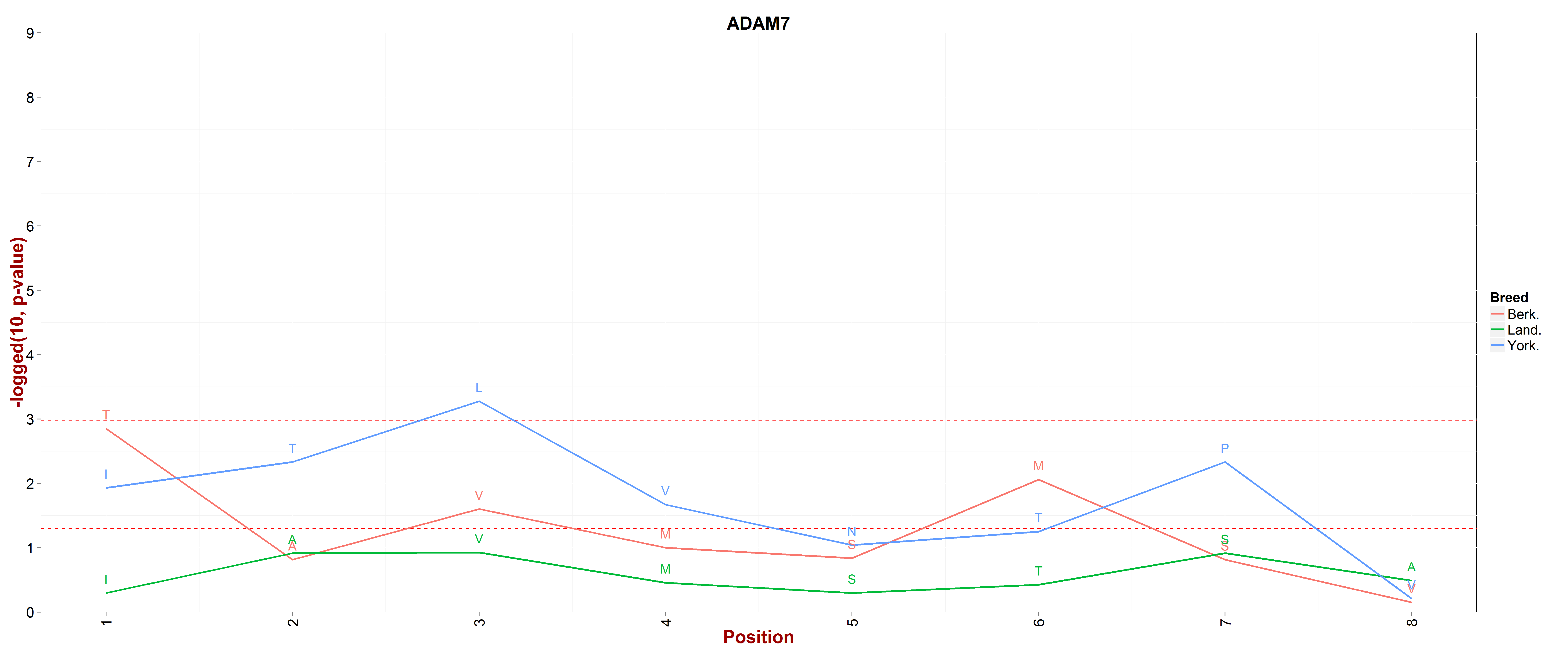


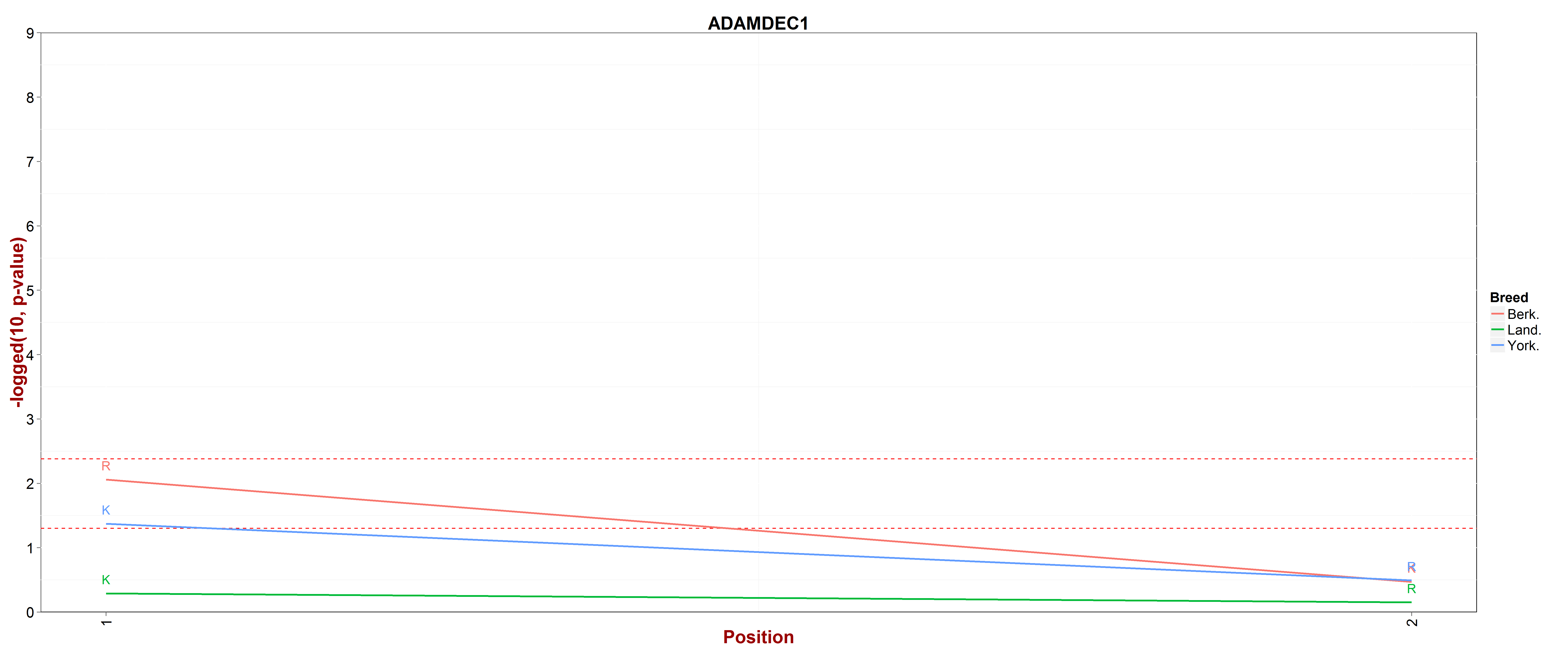

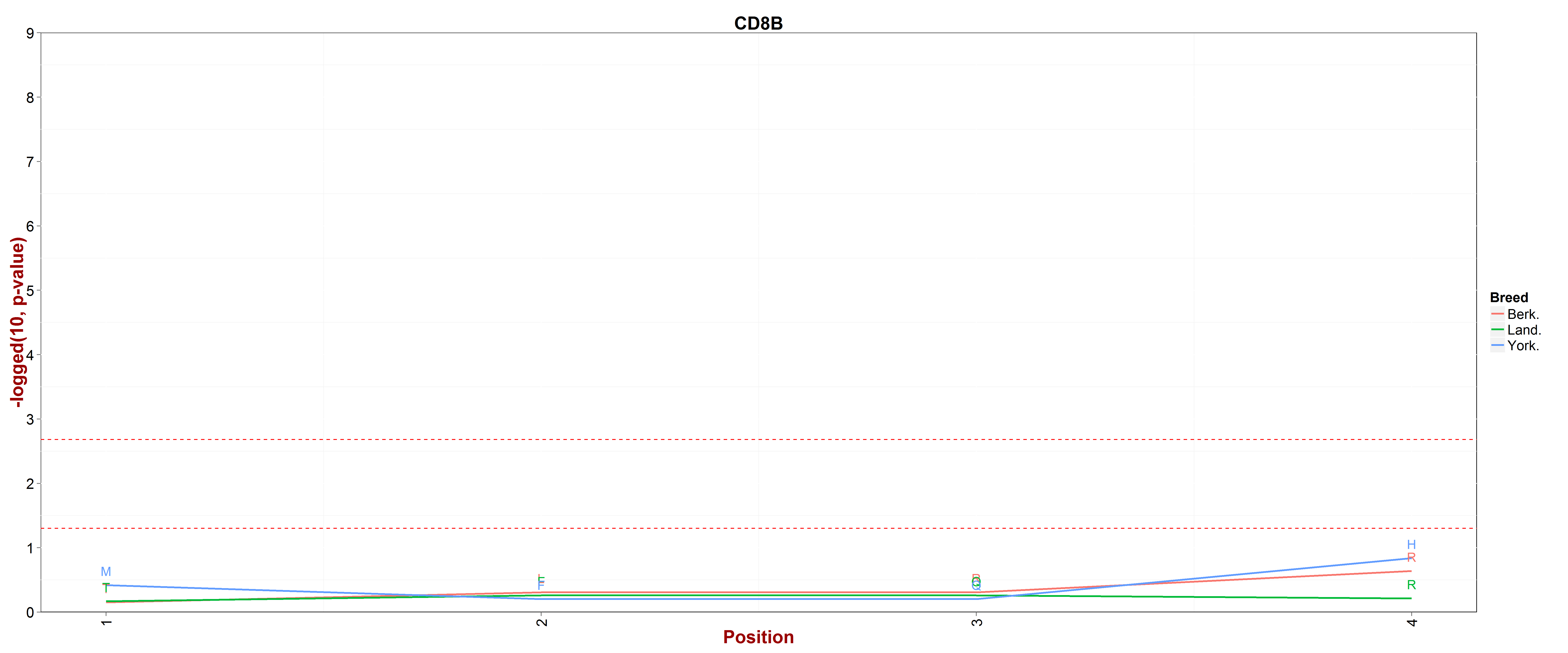


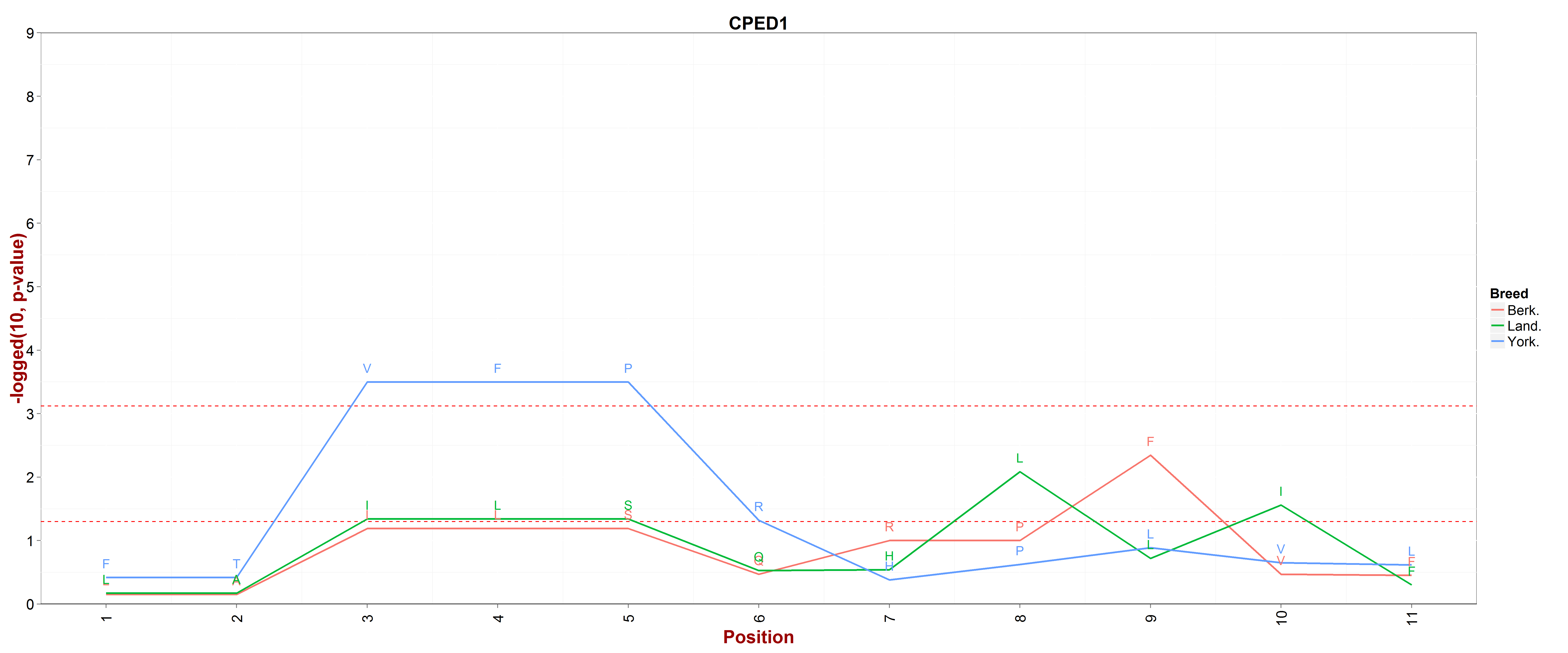


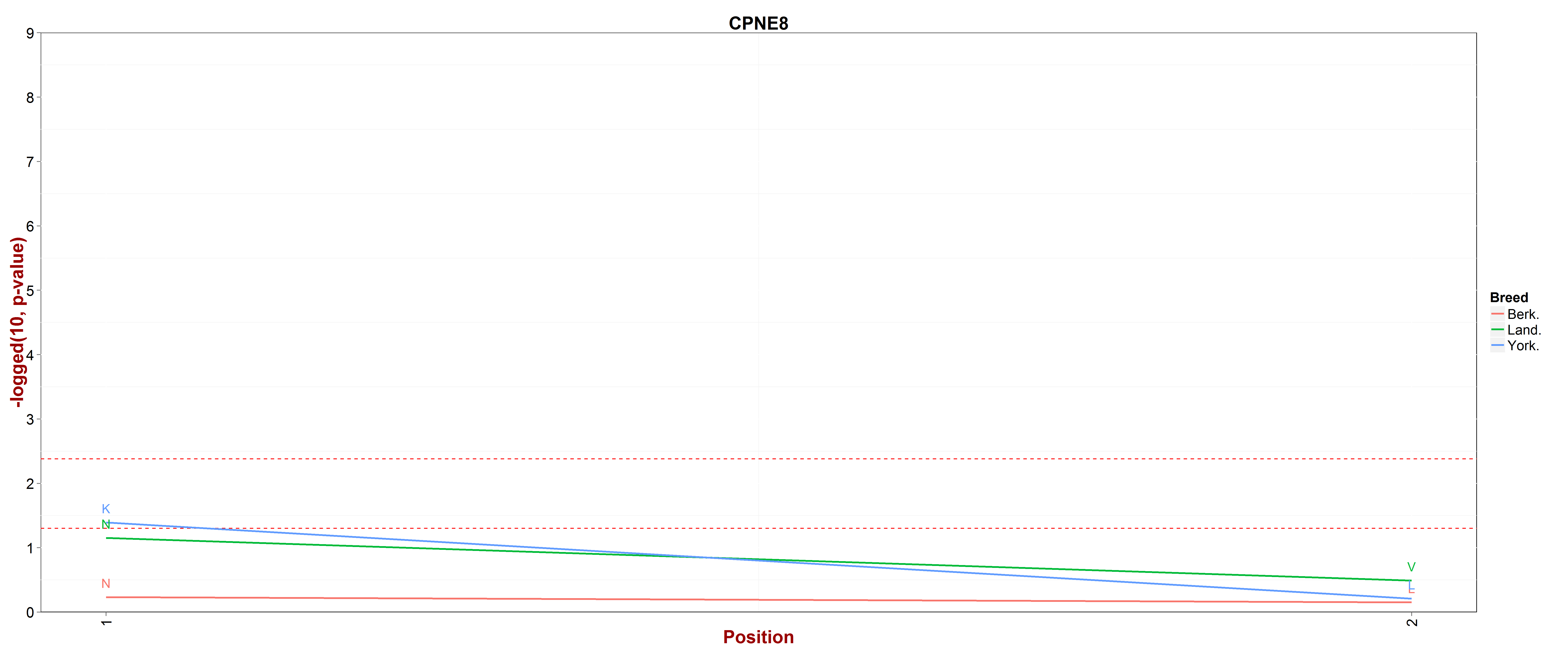


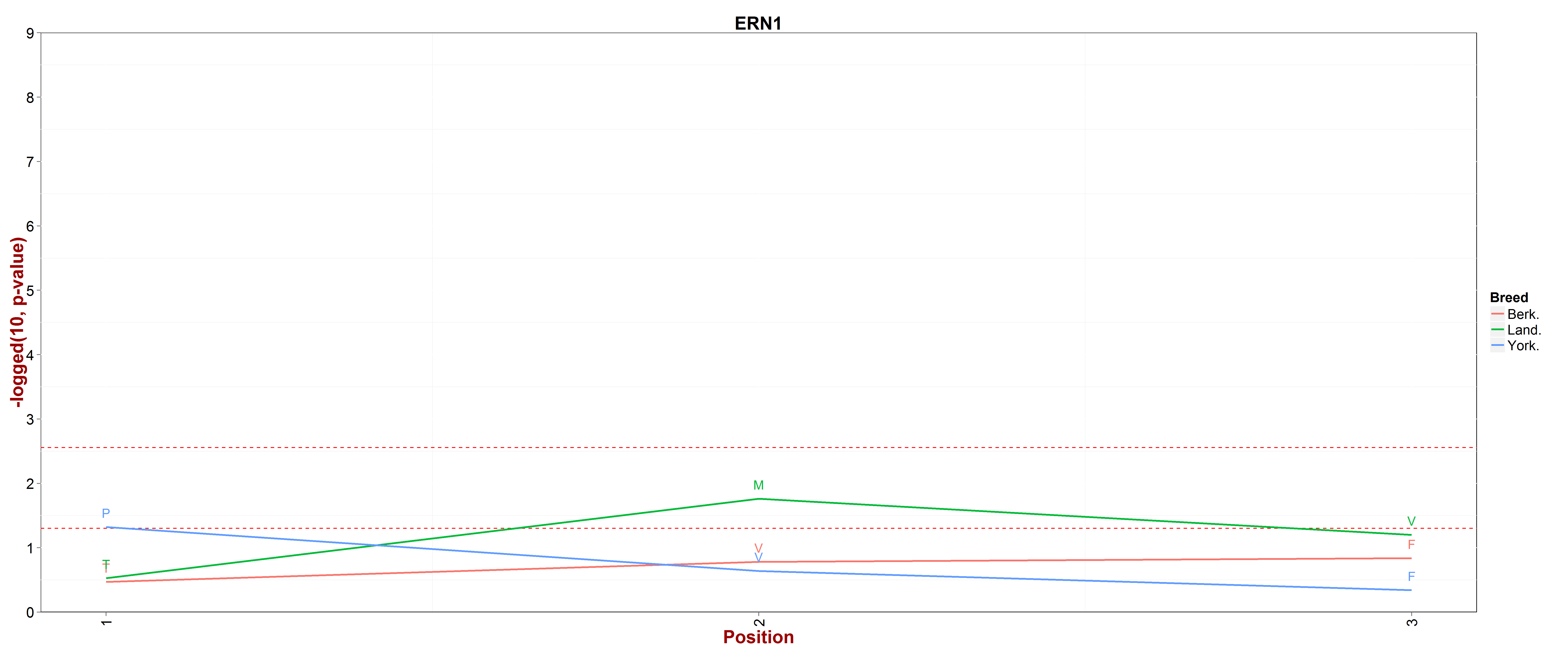


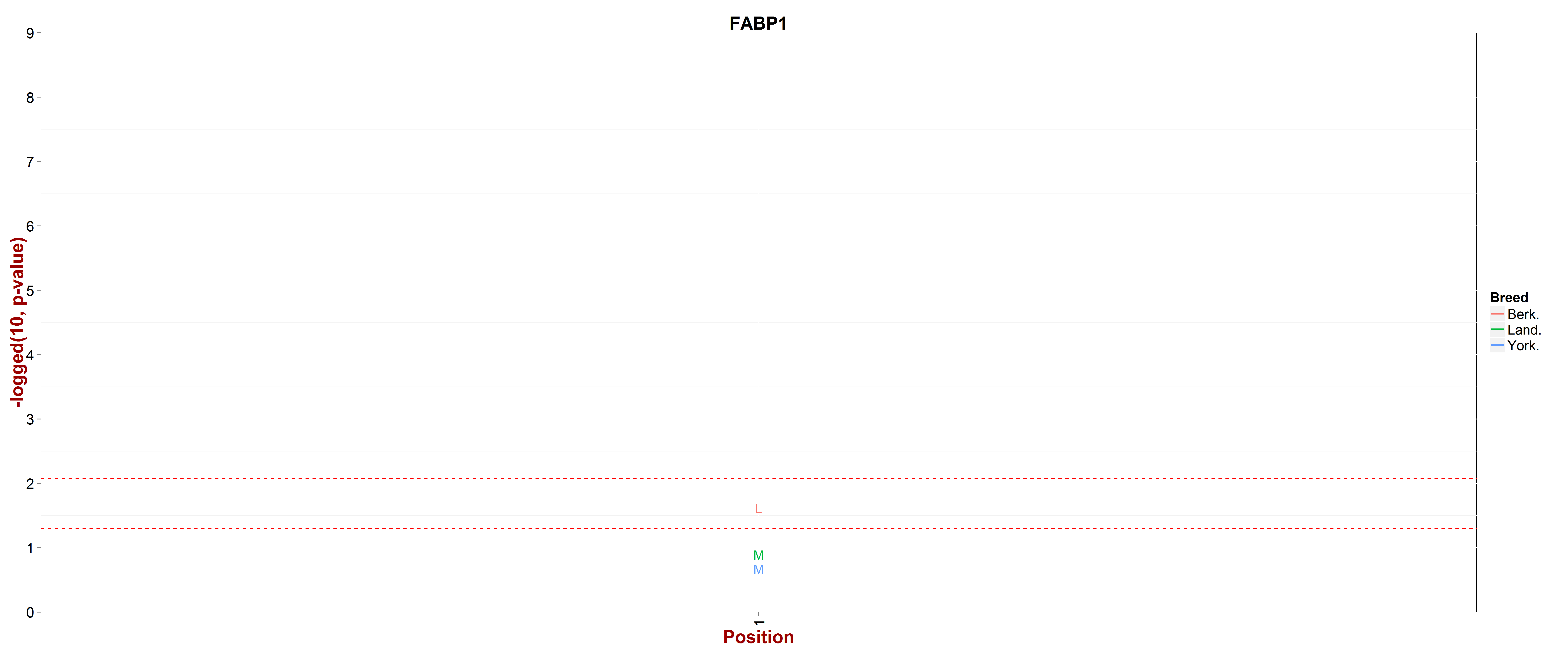


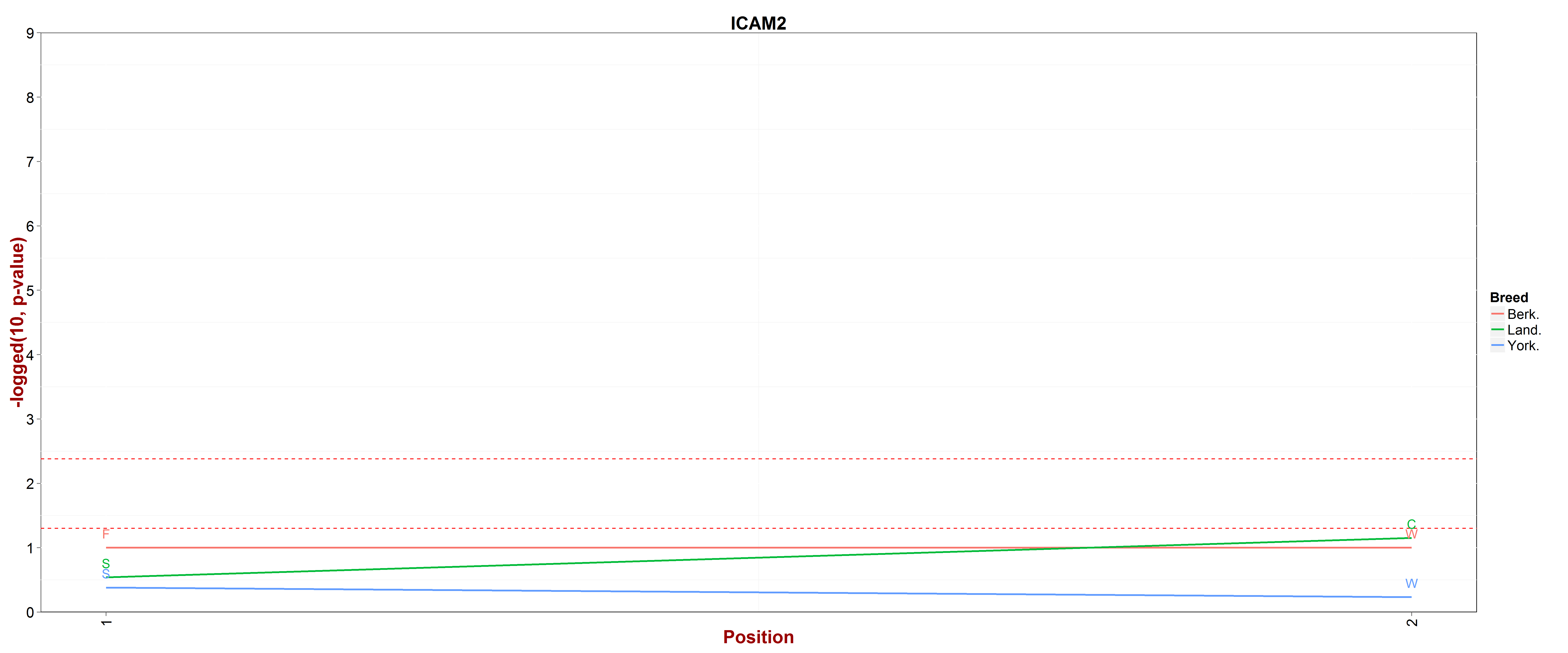


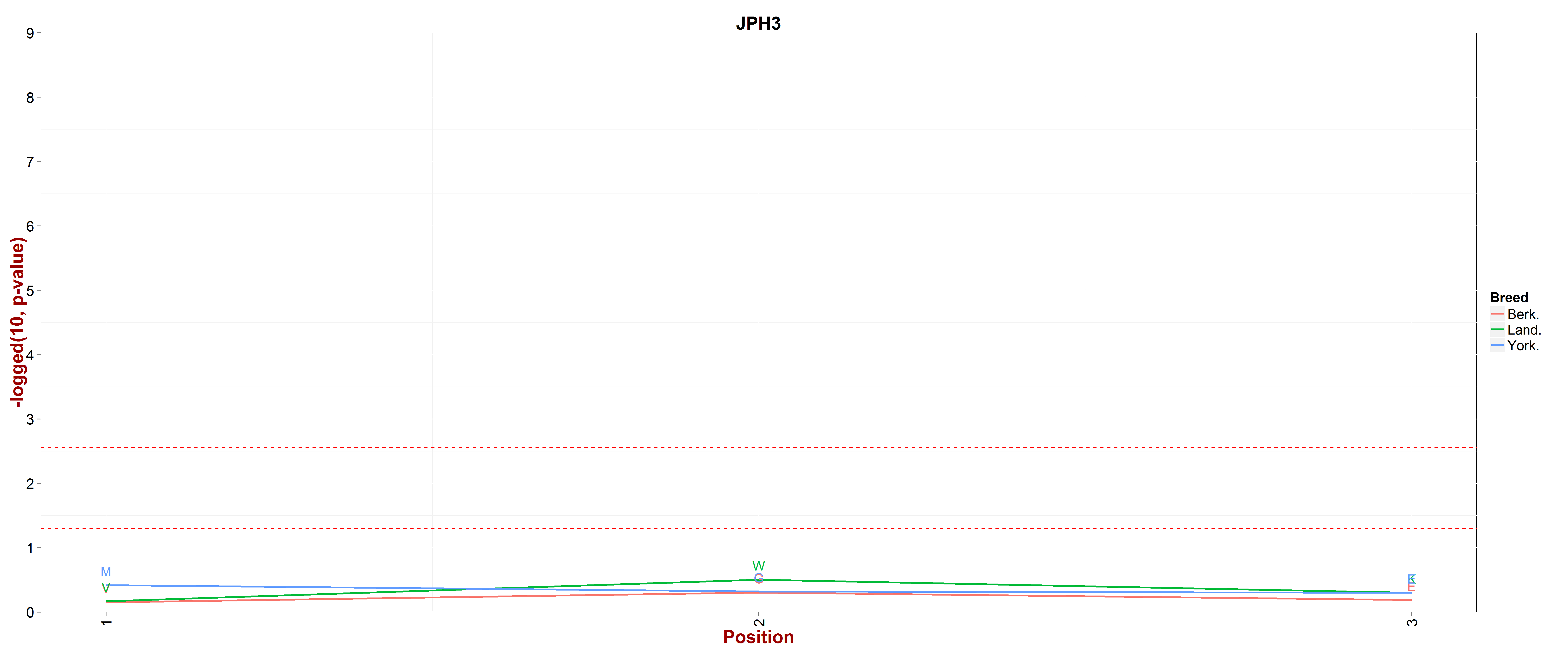


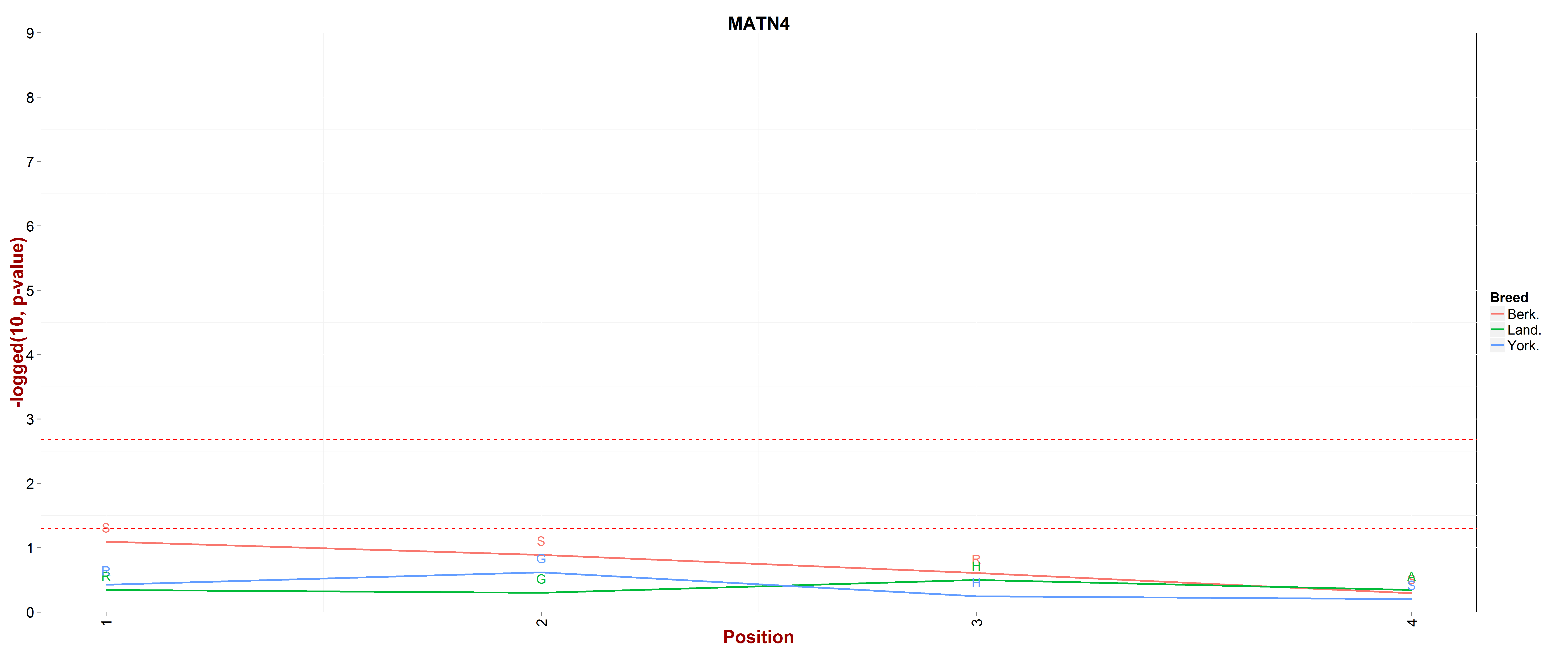


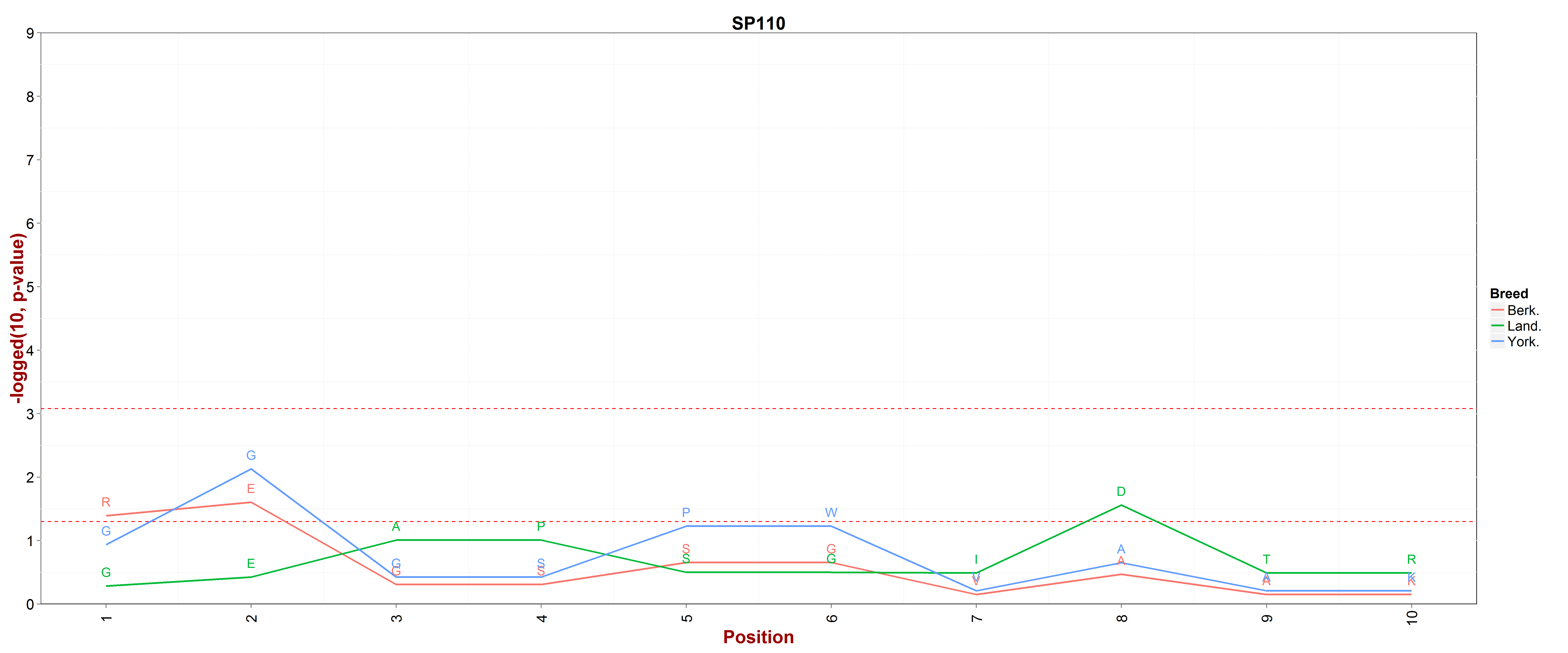


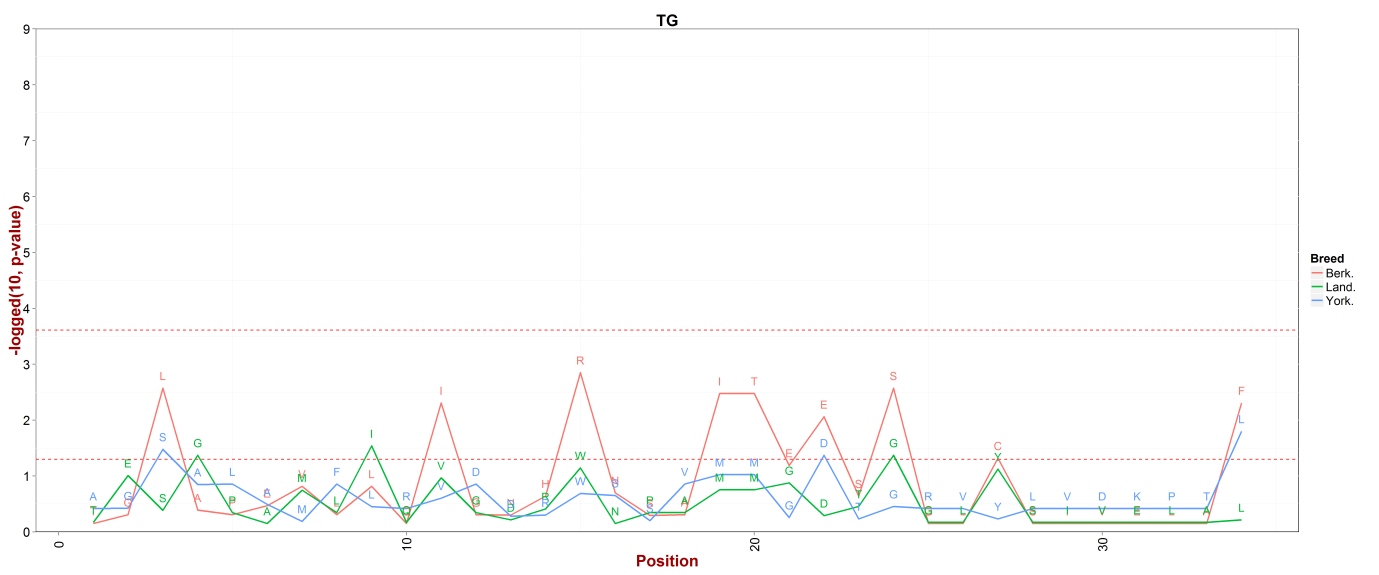


**Figure S3**. A statistical enrichment test (Fisher’s exact test) for detecting enriched Indel site on targeted genes.


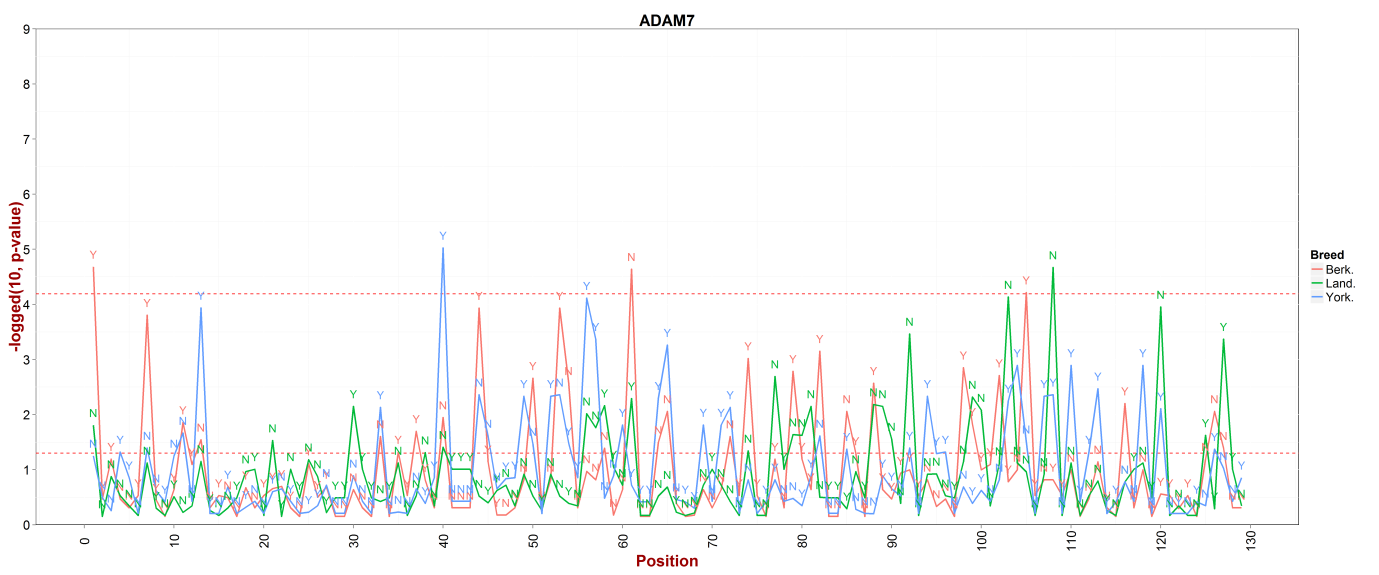


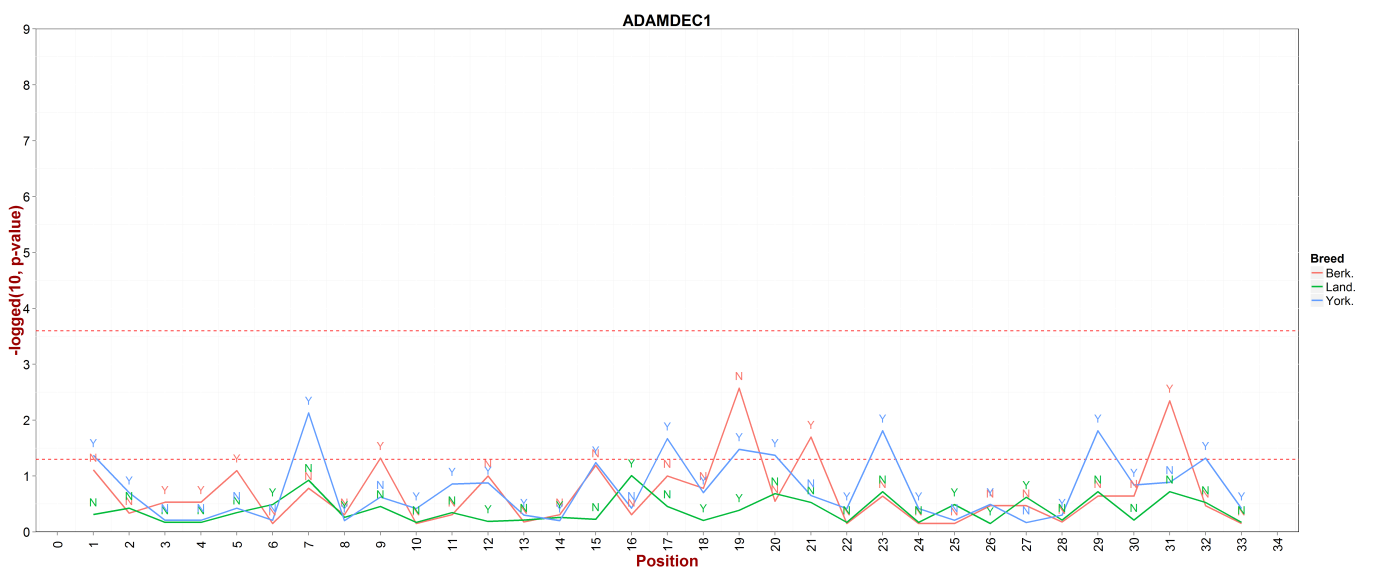

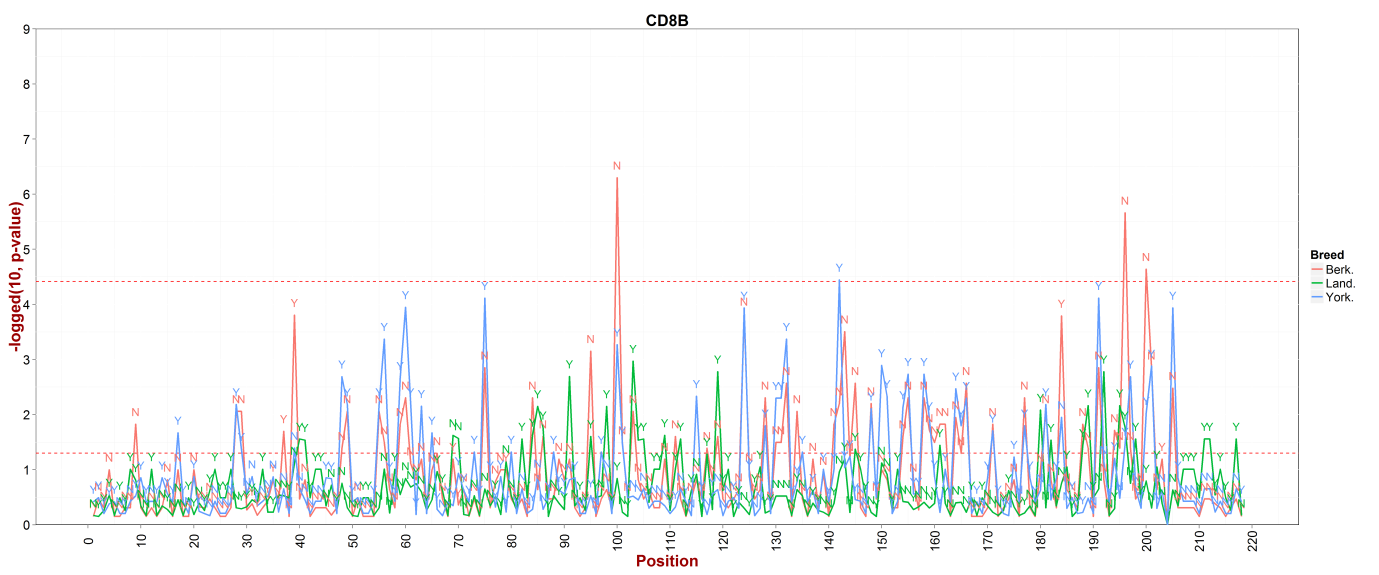


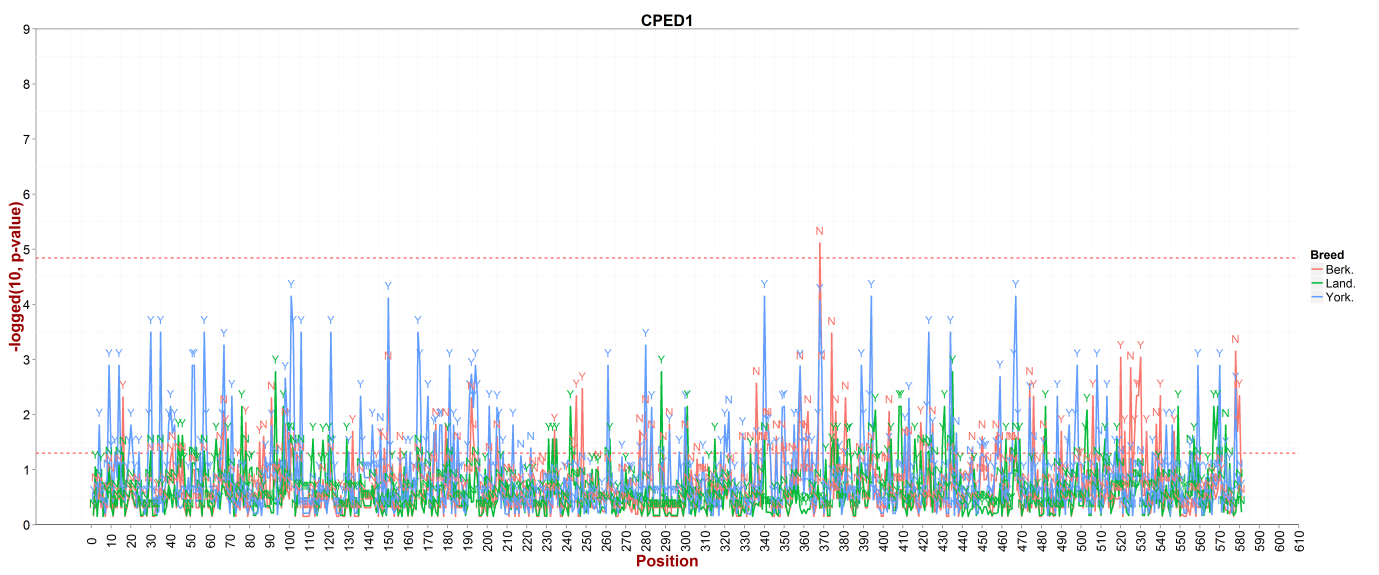


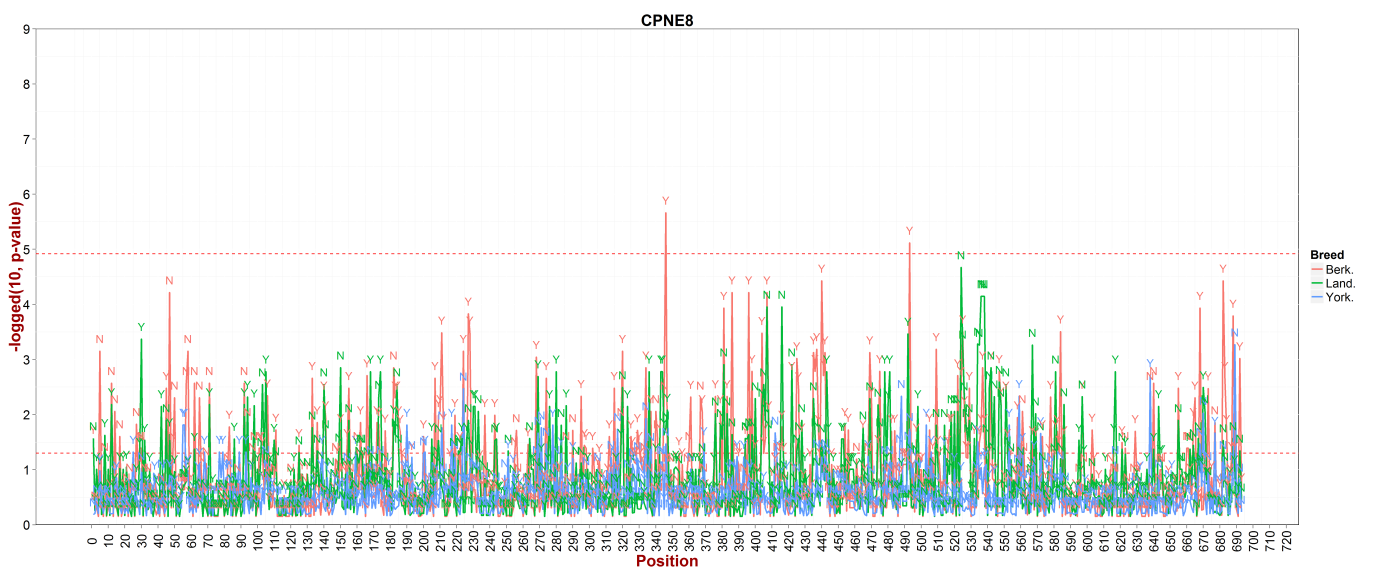


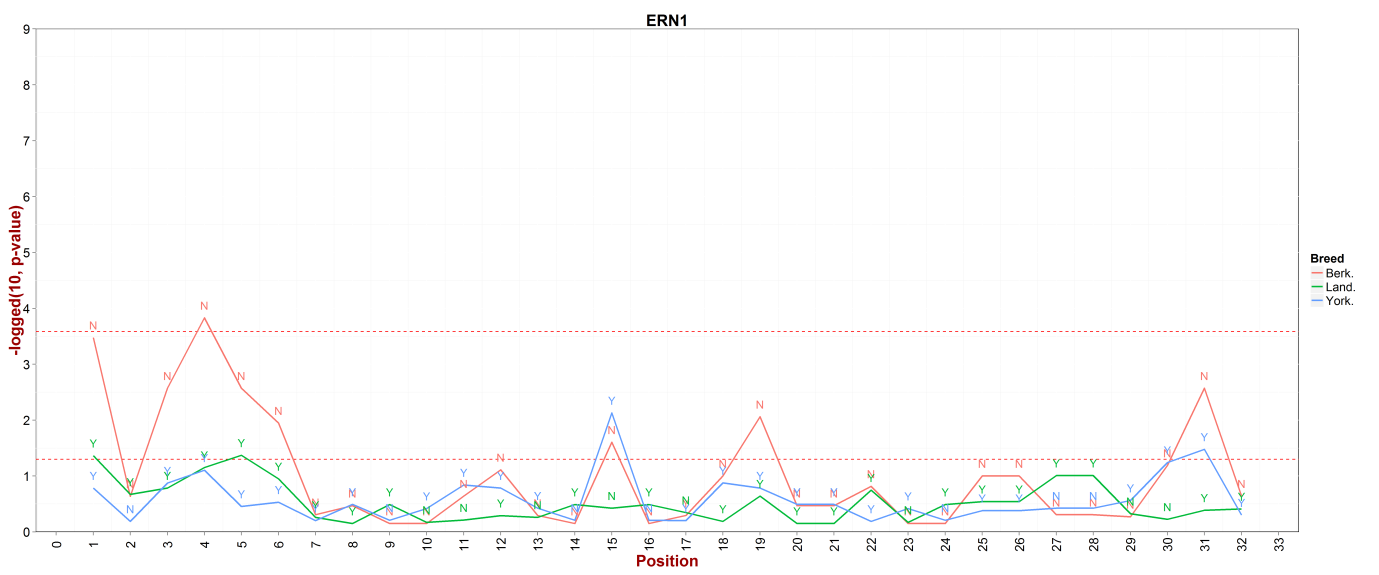


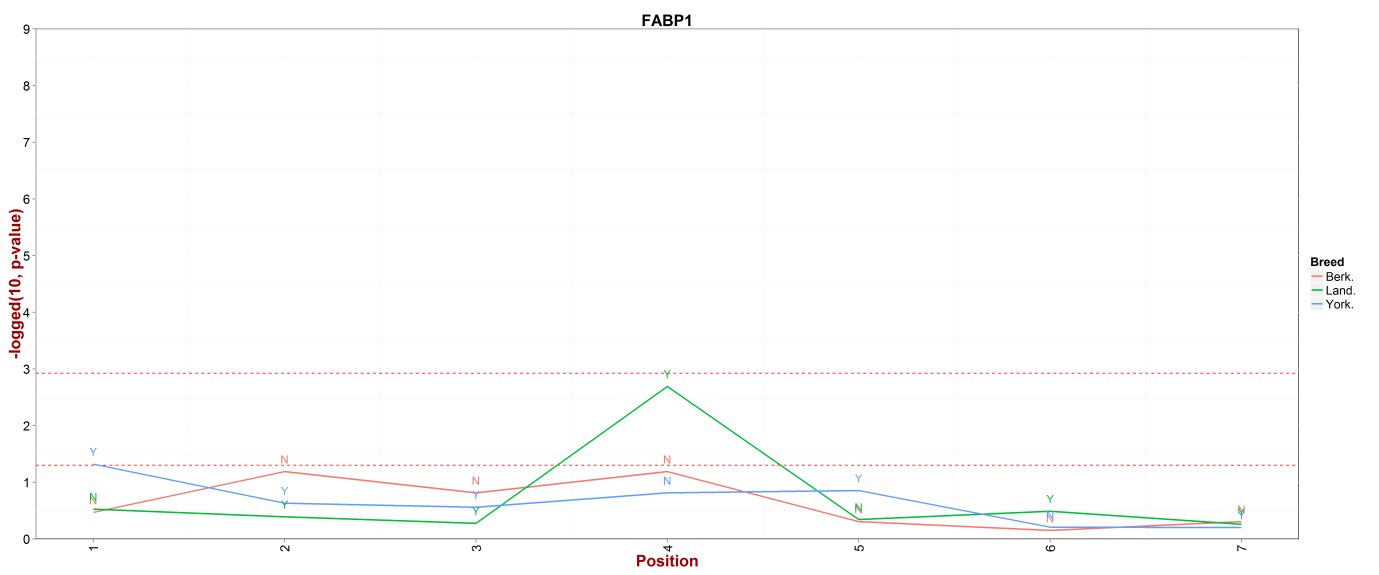


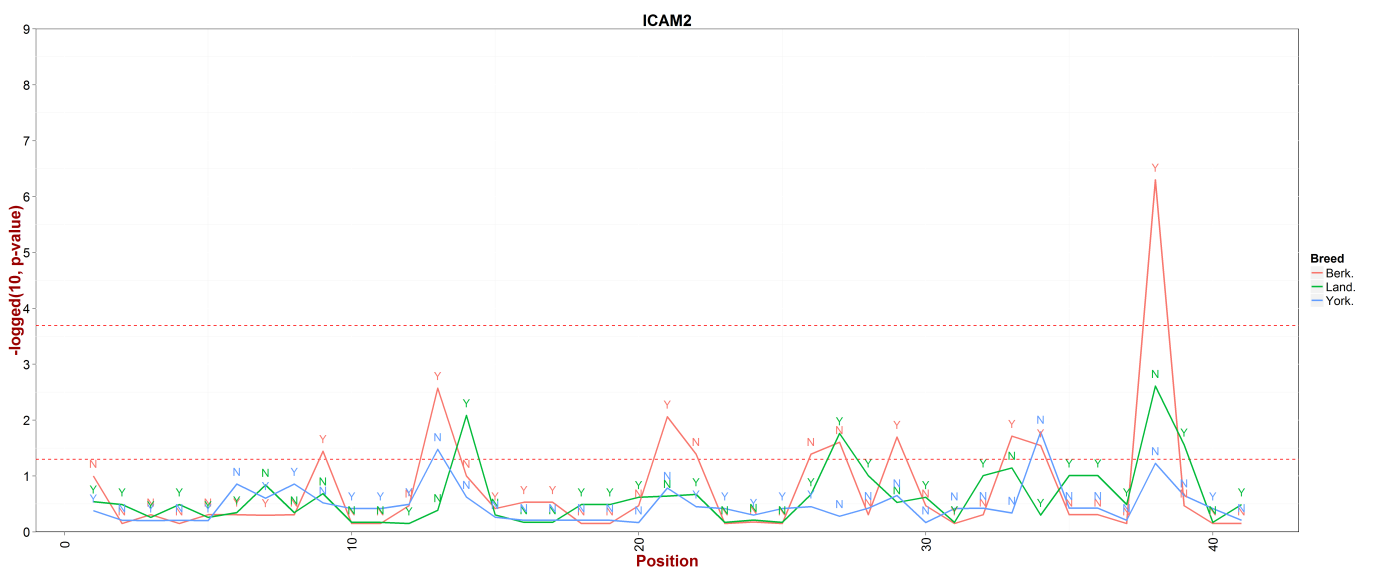


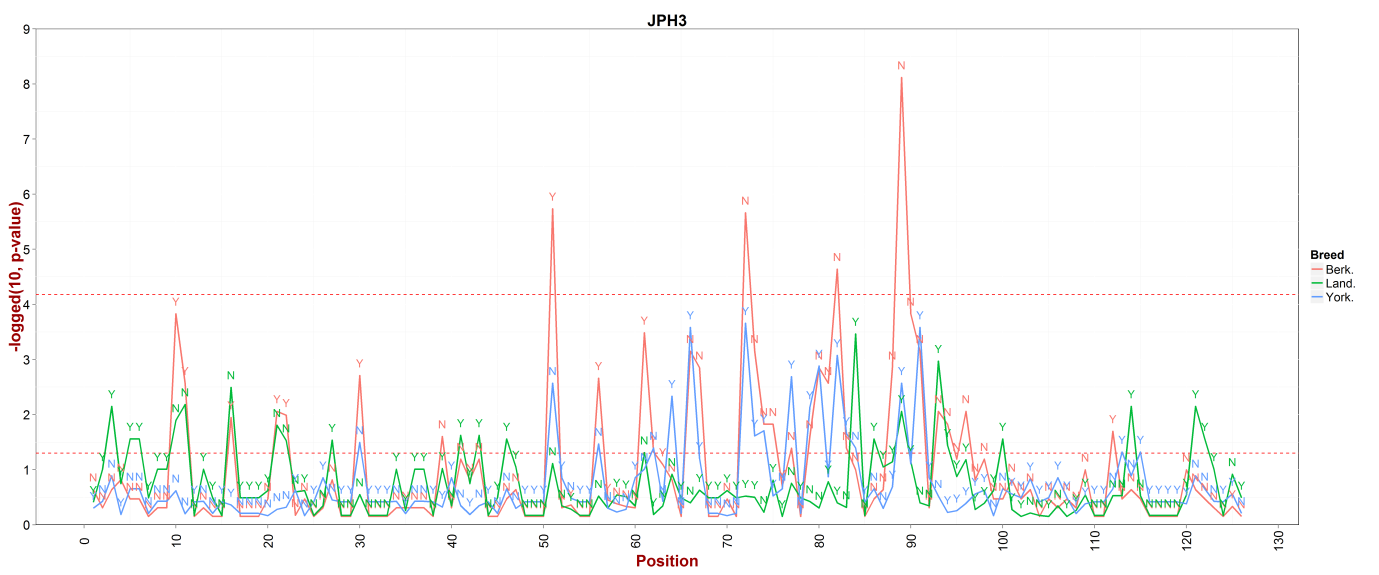


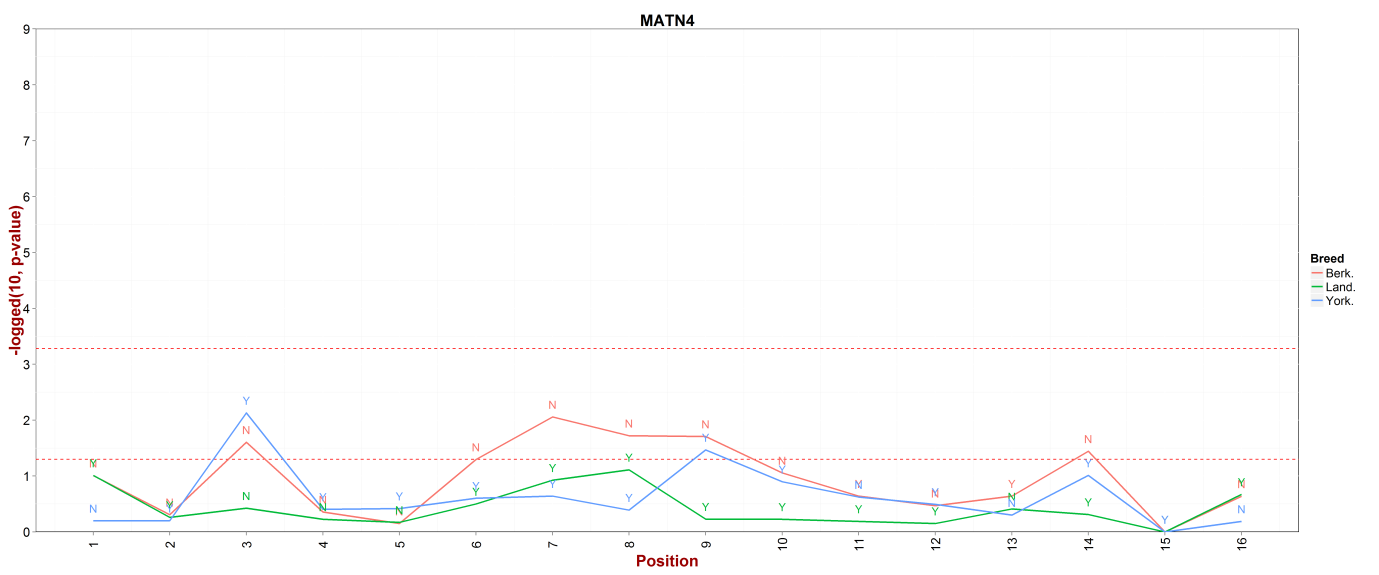


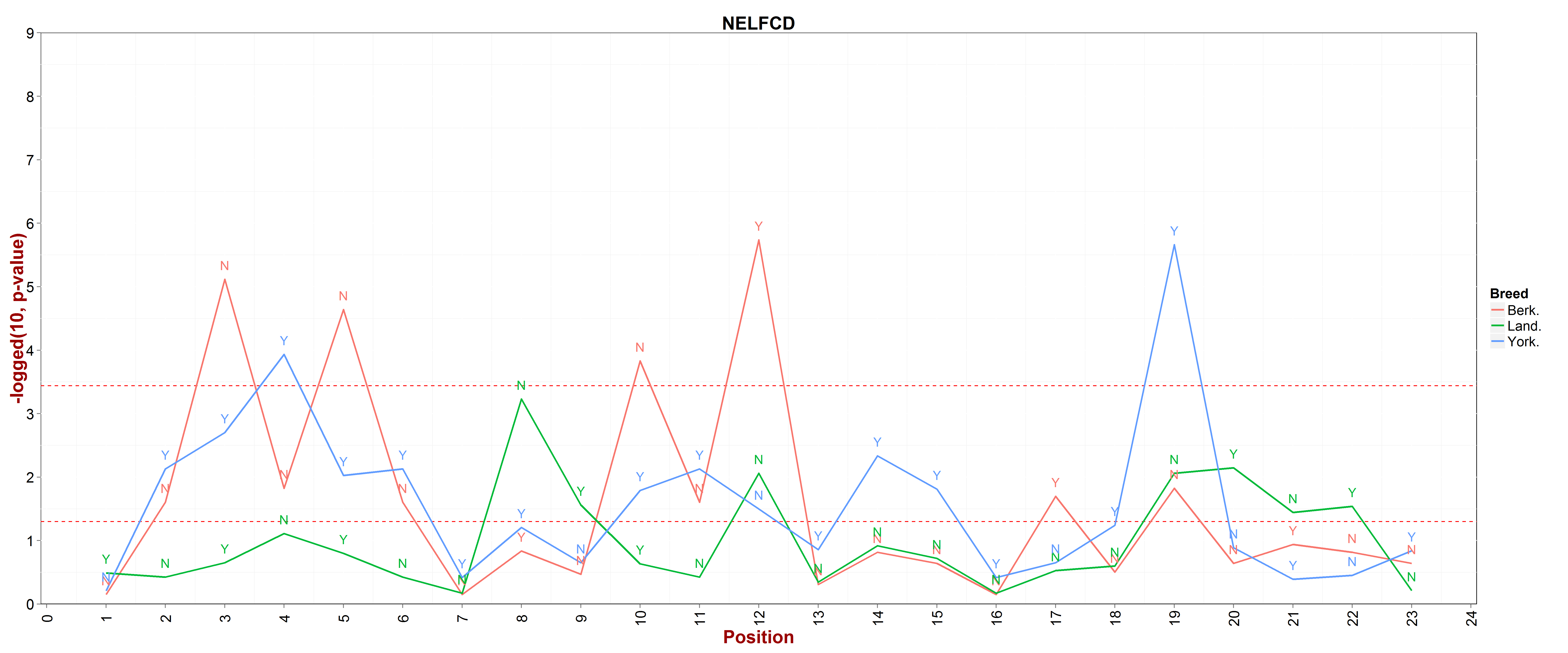


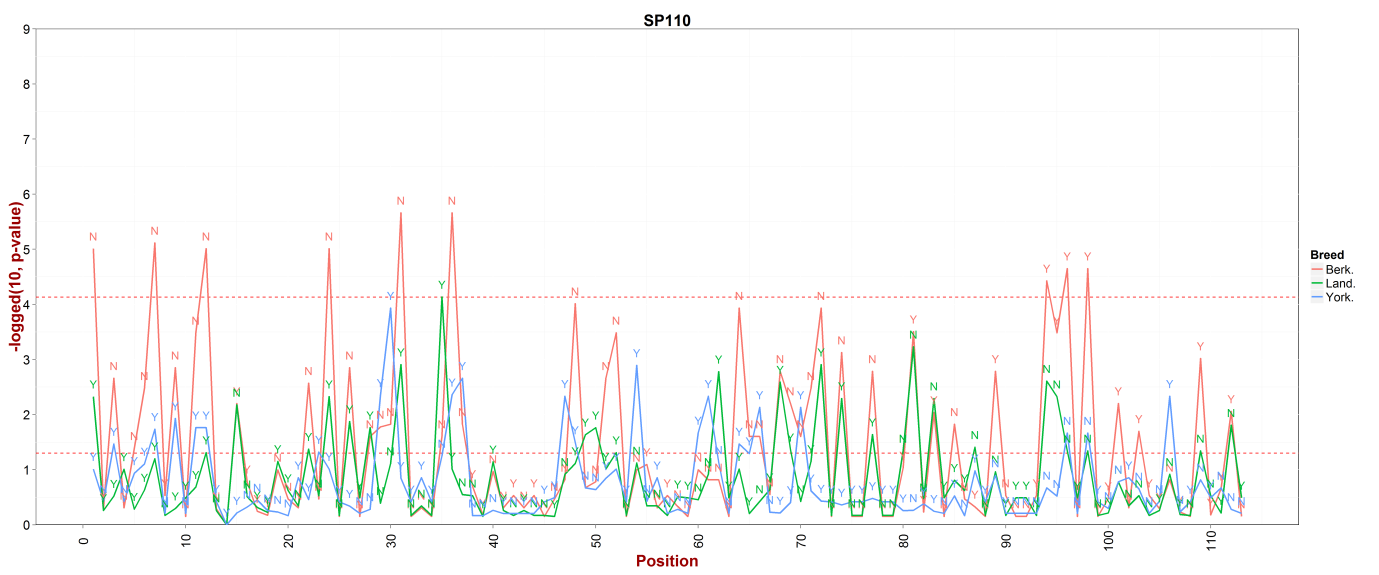


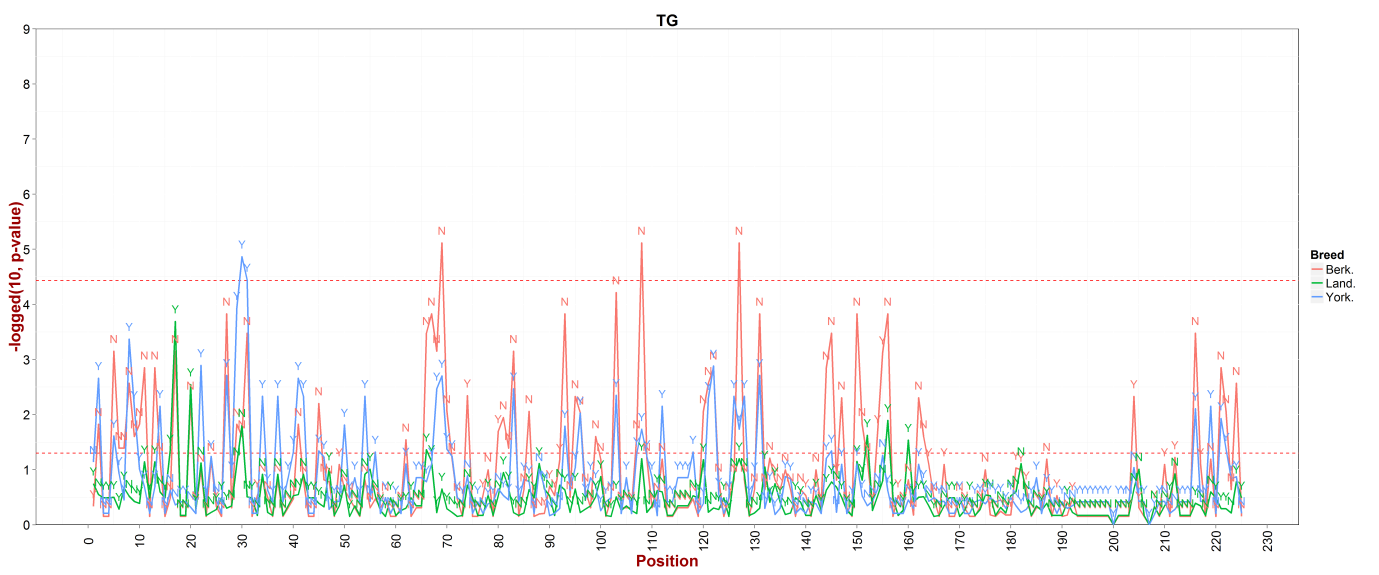


**Figure S4**. The overall reads mapping rate of assembled contigs for each breed and reference genome by aligning the total sequence reads of each Berkshire sample.


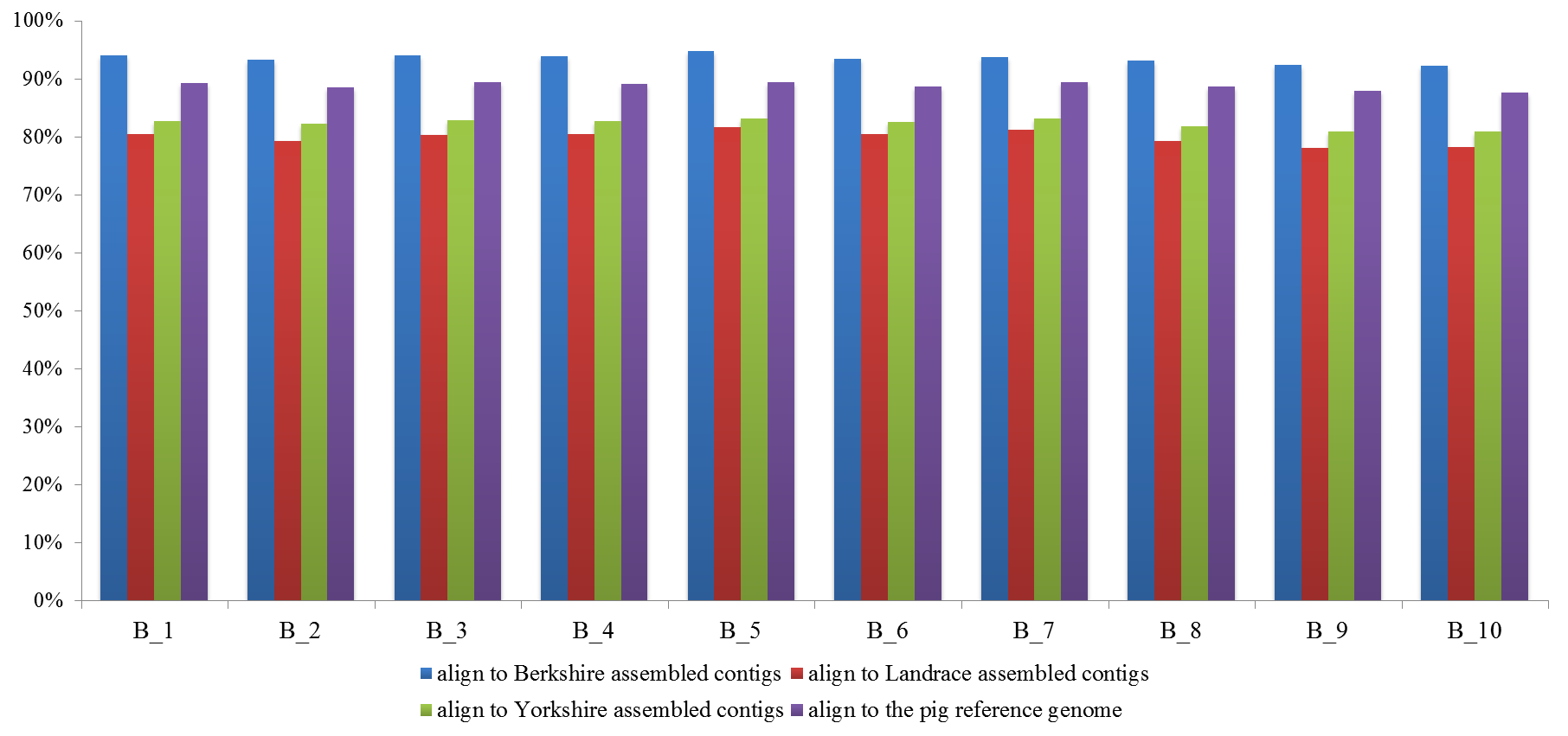

Supplement: Additional file 1: Table S1–S3. — The result summary of sequence reads mapping using Bowtie2. Table S4. List of candidate genes resulted from genome-wide positive selection scan. Table S5. Information of genes which are previously reported as meat quality related genes. Descriptions of the gene functions are based on GeneCard. Table S6. The summary statistics of assembled contigs for Berkshire, Landrace, and Yorkshire using IDBA_UD. Table S7. The result summary of assembled contigs’ repeated and transposable elements for Berkshire, Landrace, and Yorkshire; and Berkshire assembled contigs of which unmapped reads were aligned. Table S8. The alignment mapping summary of unmapped sequencing reads to the Berkshire assembled contigs. (The unmapped sequencing reads were defined as the ones that were not mapped to the reference genome and to the Landrace and Yorkshire assembled contigs.). (DOCX 61 kb) [file 12863_2015_265_MOESM1_ESM.docx]
