# Supplementary material for: Exploring evidence of positive selection reveals genetic basis of meat quality traits in Berkshire pigs through whole genome sequencing
Source: BMC Genet. 2015 Aug 20;16:104. doi: 10.1186/s12863-015-0265-1 (PMC4545873; doi:10.1186/s12863-015-0265-1)
Supplement: Additional file 2: Figure S1. — The distributions of novel SNV and known SNP in each chromosome. Figure S2. A statistical enrichment test (Fisher’s exact test) for detecting enriched non-synonymous SNP site on targeted genes. Figure S3. A statistical enrichment test (Fisher’s exact test) for detecting enriched Indel site on targeted genes. Figure S4. The overall reads mapping rate of assembled contigs for each breed and reference genome by aligning the total sequence reads of each Berkshire sample. (DOCX 3433 kb) [file 12863_2015_265_MOESM2_ESM.docx]

**Exploring evidence of positive selection reveals genetic basis of meat quality traits in Berkshire pigs through whole genome sequencing**

Authors: Hyeon Soo Jeong, Ki-Duk Song, Minseok Seo, Kelsey Caetano-Anollés, Jaemin Kim, Woori Kwak, Jae-don Oh, EuiSoo Kim, Dong Kee Jeong, Seoae Cho, Heebal Kim, Hak-Kyo Lee

| **ADDITIONAL FILE 2** |
| --- |

**Table2-9**

Table S12

Table S23

Table S34

Table S45

Table S56

Table S67

Table S78-9

Table S810

Table S1. The result summary of sequence reads mapping using Bowtie2 (Berkshire).

| Samples | Total number of reads | Paired align (concordantly) | Paired align (discordantly) | Non-paired align | Overall  alignment rate |
| --- | --- | --- | --- | --- | --- |
| B_1 | 304,804,588 (100%) | 264,655,464 (86.83%) | 1,447,350 (0.47%) | 8,018,865 (2.6%) | 89.93% |
| B_2 | 253,047,908 (100%) | 217,714,244 (86.04%) | 862,454 (0.34%) | 6,890,918 (2.72%) | 89.10% |
| B_3 | 271,420,318 (100%) | 235,657,098 (86.82%) | 983,466 (0.36%) | 7,099,349 (2.62%) | 89.80% |
| B_4 | 307,202,928 (100%) | 266,241,744 (86.67%) | 875,278 (0.28%) | 8,107,884 (2.64%) | 89.59% |
| B_5 | 443,640,008 (100%) | 386,011,330 (87.01%) | 1,795,962 (0.41%) | 11,538,051 (2.61%) | 90.02% |
| B_6 | 311,141,122 (100%) | 267,992,536 (86.13%) | 866,970 (0.28%) | 8,814,633 (2.83%) | 89.24% |
| B_7 | 288,013,648 (100%) | 249,302,488 (86.56%) | 1,297,646 (0.45%) | 8,128,979 (2.82%) | 89.83% |
| B_8 | 278,043,466 (100%) | 239,734,888 (86.22%) | 717,994 (0.26%) | 7,690,528 (2.77%) | 89.25% |
| B_9 | 263,960,864 (100%) | 224,974,736 (85.23%) | 718,198 (0.27%) | 8,020,392 (3.04%) | 88.54% |
| B_10 | 265,641,094 (100%) | 225,962,250 (85.06%) | 698,462 (0.26%) | 7,914,184 (2.98%) | 88.31% |
| Total | 2,986,915,944 (100%) | 2,578,246,778 (86.32%) | 10,263,780 (0.34%) | 82,223,783 (2.76%) | 89.36% |

* Reference pig genome: Sus_scrofa10.2
* Fastq Quality Encoding: Sanger / Illumina 1.9 encoding

Table S2. The result summary of sequence reads mapping using Bowtie2 (Landrace).

| Samples | Total number of reads | Paired align (concordantly) | Paired align (discordantly) | Non-paired align | Overall  alignment rate |
| --- | --- | --- | --- | --- | --- |
| L_1 | 327,963,242 (100%) | 284,148,166 (86.64%) | 2,759,122 (0.84%) | 8,701,697 (2.65%) | 90.13% |
| L_2 | 332,203,902 (100%) | 288,224,972 (86.76%) | 3,685,626 (1.11%) | 9,464,780 (2.85%) | 90.72% |
| L_3 | 345,708,096 (100%) | 292,140,142 (84.50%) | 9,146,420 (2.65%) | 12,172,458 (3.52%) | 90.67% |
| L_4 | 355,525,994 (100%) | 306,776,924 (86.29%) | 5,603,838 (1.58%) | 10,414,418 (2.93%) | 90.79% |
| L_5 | 335,544,768 (100%) | 287,153,812 (85.58%) | 4,133,972 (1.23%) | 10,230,992 (3.05%) | 89.86% |
| L_6 | 323,931,824 (100%) | 276,749,676 (85.43%) | 4,457,484 (1.38%) | 9,827,855 (3.03%) | 89.84% |
| L_7 | 338,310,130 (100%) | 244,994,652 (72.42%) | 22,520,534 (6.66%) | 32,642,143 (9.65%) | 88.72% |
| L_8 | 328,367,780 (100%) | 243,931,056 (74.29%) | 21,350,952 (6.50%) | 28,939,765 (8.81%) | 89.60% |
| L_9 | 301,513,518 (100%) | 167,131,020 (55.43%) | 36,254,984 (12.02%) | 49,635,358 (16.46%) | 83.92% |
| L_10 | 332,943,476 (100%) | 260,906,860 (78.36%) | 15,454,906 (4.64%) | 22,666,135 (6.81%) | 89.81% |
| L_11 | 322,384,718 (100%) | 233,801,440 (72.52%) | 18,014,646 (5.59%) | 27,801,225 (8.62%) | 86.73% |
| Total | 3,644,397,448 (100%) | 2,885,958,720 (79.19%) | 143,382,484 (3.93%) | 222,496,826 (6.11%) | 89.16% |

* Reference pig genome: Sus_scrofa10.2
* Fastq Quality Encoding: Sanger / Illumina 1.9 encoding

Table S3. The result summary of sequence reads mapping using Bowtie2 (Yorkshire).

| Samples | Total number of reads | Paired align (concordantly) | Paired align (discordantly) | Non-paired align | Overall  alignment rate |
| --- | --- | --- | --- | --- | --- |
| Y_1 | 437,075,804 (100%) | 356,548,806 (81.58%) | 1,487,336 (0.34%) | 14,373,647 (3.29%) | 85.20% |
| Y_2 | 433,671,492 (100%) | 349,459,020 (80.58%) | 1,451,608 (0.33%) | 15,270,418 (3.52%) | 84.44% |
| Y_3 | 421,654,852 (100%) | 340,456,016 (80.74%) | 2,591,788 (0.61%) | 16,919,993 (4.01%) | 85.37% |
| Y_4 | 418,666,128 (100%) | 344,925,126 (82.39%) | 1,609,106 (0.38%) | 13,636,726 (3.26%) | 86.03% |
| Y_5 | 430,293,866 (100%) | 359,466,198 (83.54%) | 1,846,106 (0.43%) | 14,002,841 (3.25%) | 87.22% |
| Y_6 | 671,036,258 (100%) | 551,036,298 (82.12%) | 5,298,938 (0.79%) | 26,208,951 (3.91%) | 86.81% |
| Y_7 | 411,119,624 (100%) | 341,692,338 (83.11%) | 1,512,428 (0.37%) | 13,201,456 (3.21%) | 86.69% |
| Y_8 | 442,784,174 (100%) | 346,812,832 (78.33%) | 1,869,420 (0.42%) | 16,987,820 (3.84%) | 82.58% |
| Y_9 | 317,653,962 (100%) | 276,538,614 (87.06%) | 2,637,236 (0.83%) | 8,945,027 (2.82%) | 90.70% |
| Y_10 | 339,820,584 (100%) | 287,058,268 (84.47%) | 7,002,276 (2.06%) | 11,034,708 (3.25%) | 89.78% |
| Y_11 | 351,288,112 (100%) | 297,061,500 (84.56%) | 7,087,374 (2.02%) | 12,323,309 (3.51%) | 90.09% |
| Y_12 | 341,544,022 (100%) | 269,254,168 (78.83%) | 21,493,938 (6.29%) | 15,690,652 (4.59%) | 89.72% |
| Y_13 | 333,812,260 (100%) | 277,778,240 (83.21%) | 12,344,680 (3.70%) | 12,871,050 (3.86%) | 90.77% |
| Total | 5,350,421,138 (100%) | 4,398,087,424 (82.20%) | 68,232,234 (1.28%) | 191,466,598 (3.58%) | 87.34% |

* Reference pig genome: Sus_scrofa10.2
* Fastq Quality Encoding: Sanger / Illumina 1.9 encoding

Table S4. List of candidate genes resulted from genome-wide positive selection scan (overlapped in Berkshire-Yorkshire and Berkshire-Landrace).

¶Genes within regions resulted from XP-EHH.

*Genes within regions resulted from XP-CLR.

*∮*Genes within regions resulted from both XP-EHH and XP-CLR.

*5S_rRNA **

*7SK ∮*

*ABCA13 **

*ABI1 **

*ACTN2 ¶*

*ADAM7 ∮*

*ADAMDEC1 ∮*

*ADPRM **

*AKIRIN2 ¶*

*AMPH **

*ANKRD16 **

*APBB1IP **

*ASB13 **

*ATP5E **

*ATP9A **

*ATXN7L3B ¶*

*BACH2 **

*BCAP29 **

*BIN2 **

*BTBD9 ¶*

*C2CD3 ¶*

*CCDC30 **

*CCR2 ¶*

*CCR5 ¶*

*CCRL2 ¶*

*CD79B **

*CD8B ∮*

*CEP152 ¶*

*COL5A1 **

*CPED1 ∮*

*CPNE8 ∮*

*CTSZ **

*CU469018.1 **

*CUL1 ¶*

*CWC15 **

*DAZAP2 **

*DUS4L **

*ELAC2 ¶*

*ELP6 ¶*

*ENOX1 ¶*

*ERN1 ∮*

*FABP1 ∮*

*FAM114A2 **

*FAM134B **

*FAM208B **

*FBXO18 **

*FBXO31 **

*FLT3 **

*FNDC8 **

*FRAS1 ¶*

*FRMD4B **

*GABRR2 **

*GDI2 **

*GH **

*GLP2R ¶*

*GNAS **

*GPATCH2 **

*GPR111 **

*ICAM2 ∮*

*IFNGR1 **

*IGKV-5 ¶*

*IGKV-6 ¶*

*IGKV-7 ¶*

*IGSF5 **

*IKBKAP **

*IL22RA2 **

*IL2RA **

*INTS4 **

*ITGA1 ¶*

*JPH3 ∮*

*KCND2 ¶*

*KIAA0100 ¶*

*KIAA1009 **

*KLHL1 **

*KRCC1 **

*LDB2 **

*LPHN3 ¶*

*LRRC6 **

*MAP1LC3B **

*MARCH10 **

*MATN4 ∮*

*MEP1A **

*MRAP2 **

*MRC2 **

*MRPL1 ¶*

*MTSS1 **

*MTUS2 **

*MYO10 **

*NELFCD ∮*

*NFATC2 **

*NLE1 **

*NPEPL1 ¶*

*NUP62 ¶*

*OLFM1 **

*OLFML1 **

*OR52E4 ¶*

*OR52N1 **

*OR52N5 **

*ORC3 ¶*

*OTOGL **

*OVCH2 **

*P2X3R **

*PDIA4 **

*PHF20L1 **

*POLA2 **

*PPME1 ¶*

*PPP2R5C ¶*

*PROM1 **

*RAE1 **

*RARS2 ¶*

*RBM17 **

*RCAN2 **

*RFFL **

*RGS18 ¶*

*RIMKLA **

*RMND5A **

*RNF4 **

*RNPC1 **

*RRP1B ¶*

*SCAP ¶*

*SCN4A **

*SCO1 **

*SLMO2 **

*SLPI **

*SMAGP **

*SMYD1 **

*SNORA70 **

*SP110 ∮*

*SPAG4L **

*SPAG5 ¶*

*SRSF12 ¶*

*SYT1 **

*SZT2 **

*TG ∮*

*TGFBR3 ¶*

*TIMP-2 **

*TMEM220 **

*TNFRSF19 **

*TNFRSF21 **

*TRAPPC9 **

*TUBB1 **

*TUBGCP3 **

*U6 ∮*

*UBE2J1 **

*UNC45B **

*USP25 ¶*

*USP36 **

*XCR1 ¶*

*Y_RNA **

*ZBP1 **

*ZC3HAV1L **

*ZMYND12 **

*ssc-mir-296 ¶*

*ssc-mir-30b ¶*

*ssc-mir-30d ¶*

Table S5. Information of genes which are previously reported as meat quality related genes. Descriptions of the gene functions are based on GeneCard [[1](#_ENREF_1)].

| Gene | Function | Reference |
| --- | --- | --- |
| *TG* | Acts as a substrate for the synthesis of thyroxine and iodothyronine as well as the storage of the inactive forms of thyroid hormone and iodine. | [[2-5](#_ENREF_2)] |
| *FABP1* | Encodes the fatty acid binding protein found in liver. FABPs roles include fatty acid uptake, transport, and metabolism. | [[6-9](#_ENREF_6)] |
| *AKIRIN2* | Required for the innate immune response and response to lipopolysaccharide. | [[10](#_ENREF_10)] |
| *GLP2R* | Stimulates intestinal growth and upregulates villus height in the small intestine, concomitant with increased crypt cell proliferation and decreased enterocyte apoptosis. | [[11](#_ENREF_11)] |
| *TGFBR3* | Involved in capturing and retaining TGF-beta for presentation to the signaling receptors. | [[12](#_ENREF_12), [13](#_ENREF_13)] |
| *JPH3* | Contributes to the formation of junctional membrane complexes (JMCs) which link the plasma membrane with the endoplasmic or sarcoplasmic reticulum in excitable cells. | [[14](#_ENREF_14)] |
| *ERN1* | ERN1 is a transmembrane resident ER protein that possesses both kinase and endonuclease domains | [[15](#_ENREF_15)] |
| *SLC25A14* | Reduces the mitochondrial membrane potential in mammalian cells, and is widely expressed in many tissues with the greatest abundance in brain and testis. | [[16](#_ENREF_16)] |
| *IGF1* | Similar to insulin in function and structure and is a member of a family of proteins involved in mediating growth and development. | [[17](#_ENREF_17)] |
| *PI4KA* | Encodes a phosphatidylinositol (PI) 4-kinase which catalyzes the first committed step in the biosynthesis of phosphatidylinositol 4,5-bisphosphate. | [[18](#_ENREF_18)] |
| *CACNA1A* | Mediates the entry of calcium ions into excitable cells and muscle contraction, hormone or neurotransmitter release. | [[19](#_ENREF_19)] |

Table S6. The summary statistics of assembled contigs for Berkshire, Landrace, and Yorkshire using IDBA_UD.

| **Sample name** | **Berkshire assembled contigs** | **Landrace assembled contgs** | **Yorkshire assembled contigs** |
| --- | --- | --- | --- |
| **Number of contigs** | 127,713 | 270,296 | 271,102 |
| **Sequence lengths** |  |  |  |
| Minimum length | 2,000 | 2,000 | 2,000 |
| Maximum length | 376,136 | 100,559 | 121,658 |
| Average length | 18,040 | 7,130 | 7,363 |
| N50 length | 30,158 | 9,379 | 9,695 |
| **Residue contents** |  |  |  |
| GC contents (%) | 2,303,986,880 | 1,927,202,658 | 1,995,955,586 |
| Total residue counts (bp) | 2,303,986,880 | 1,927,202,658 | 1,995,955,586 |
| N contents | 3,994,159 | 12,552,162 | 11,987,309 |
| Closed N by Gapcloser | 3,683,387 | 4,939,252 | 4,652,510 |

Table S7. The result summary of assembled contigs’ repeated and transposable elements for Berkshire, Landrace, and Yorkshire; and Berkshire assembled contigs of which unmapped reads were aligned.

| **Sample name** | **Berkshire  assembled contigs** | **Landrace  assembled contgs** | **Yorkshire  assembled contigs** | **Berkshire assembled contigs  (aligned by unmapped reads)** |
| --- | --- | --- | --- | --- |
| **SINE elements** | 327,645,056 (14.2%) | 250,638,900 (13%) | 271,366,754 (13.6%) | 210,150 (8.9%) |
| MIRs | 51,816,119 (2.3%) | 43,089,186 (2.2%) | 44,987,615 (2.3%) | 30,034 (1.3%) |
| **LINE elements** | 438,514,901 (19%) | 374,311,562 (19.4%) | 371,221,247 (18.6%) | 266,331 (11.3%) |
| LINE1 | 372,585,878 (16.17%) | 319,598,700 (16.6%) | 314,801,771 (15.8%) | 238,532 (10.1%) |
| LINE2 | 57,265,899 (2.5%) | 47,373,311 (2.5%) | 49,015,357 (2.5%) | 25,375 (1.1%) |
| L3/CR1 | 6,129,792 (0.3%) | 5,049,159 (0.3%) | 5,108,792 (0.3%) | 1,819 (0.1%) |
| RTE | 2,389,095 (0.1%) | 2,161,378 (0.1%) | 2,168,000 (0.1%) | 605 (0.03%) |
| **LTR elements** | 111,174,277 (4.8%) | 99,511,787 (5.2%) | 102,484,633 (5.1%) | 79,486 (3.4%) |
| ERVL | 31,960,426 (1.4%) | 29,152,605 (1.5%) | 29,872,332 (1.5%) | 21,059 (0.9%) |
| ERVL-MaLRs | 42,664,740 (1.9%) | 38,309,685 (2%) | 39,598,614 (2%) | 23,706 (1.0%) |
| ERV_class I | 29,861,951 (1.3%) | 26,117,756 (1.4%) | 27,085,634 (1.4%) | 29,499 (1.3%) |
| ERV_class II | 2,185,840 (0.1%) | 1,763,769 (0.1%) | 1,734,959 (0.1%) | 3,209 (0.2%) |
| **DNA elements** | 58,177,227 (2.5%) | 50,554,422 (2.6%) | 51,582,476 (2.6%) | 29,549 (1.3%) |
| hAT-Charlie | 31,265,469 (1.4%) | 26,880,273 (1.4%) | 27,613,550 (1.4%) | 20,302 (0.9%) |
| TcMar-Tigger | 12,426,706 (0.5%) | 11,178,616 (0.6%) | 11,242,173 (0.6%) | 4,049 (0.2%) |
| **Unclassified** | 920,632 (0.04%) | 850,145 (0.04%) | 855,520 (0.04%) | 530 (0.02%) |
| **Small RNA** | 275,807,479 (12%) | 207,554,004 (10.8%) | 226,388,127 (11.3%) | 180,478 (7.6%) |
| **Satellites** | 2,084,392 (0.1%) | 1,397,379 (0.06%) | 1,466,892 (0.06%) | 577,725 (24.4%) |
| Total bases masked | 938,893,022 (40.8%) | 777,586,827 (40.4%) | 799,311,123 (40.1%) | 1,160,831 (49.1%) |

Table S8. The alignment mapping summary of unmapped sequencing reads to the Berkshire assembled contigs. (The unmapped sequencing reads were defined as the ones that were not mapped to the reference genome and to the Landrace and Yorkshire assembled contigs.)

| **Categories** | **Samples** | | | | | | | | | |
| --- | --- | --- | --- | --- | --- | --- | --- | --- | --- | --- |
|  | **B_1** | **B_2** | **B_3** | **B_4** | **B_5** | **B_6** | **B_7** | **B_8** | **B_9** | **B_10** |
| Total number of reads | 13,628,433 (100%) | 8,368,548 (100%) | 10,681,941 (100%) | 10,847,541 (100%) | 12,304,001 (100%) | 8,479,291 (100%) | 8,013,873 (100%) | 9,436,249 (100%) | 10,525,985 (100%) | 8,293,606 (100%) |
| Aligned 0 time | 8,471,987 (62.16%) | 4,814,337 (57.53%) | 7,139,625 (66.84%) | 7,098,207 (65.44%) | 8,686,418 (70.60%) | 4,819,690 (56.84%) | 4,607,244 (57.49%) | 5,588,090 (59.22%) | 6,743,788 (64.07%) | 5,094,468 (61.43%) |
| Aligned exactly 1 time | 4,662,069 (34.21%) | 3,108,536 (37.15%) | 3,165,728 (29.64%) | 3,325,796 (30.66%) | 3,207,814 (26.07%) | 3,291,476 (38.82%) | 3,041,792 (37.96%) | 3,379,386 (35.81%) | 3,380,408 (32.11%) | 2,814,182 (33.93%) |
| Aligned more than 1 time | 494,377 (3.63%) | 445,675 (5.33%) | 376,588 (3.53%) | 423,538 (3.90%) | 409,769 (3.33%) | 368,125 (4.34%) | 364,837 (4.55%) | 468,773 (4.97%) | 401,789 (3.82%) | 384,956 (4.64%) |
| Overall alignment rate | 37.84% | 42.47% | 33.16% | 34.56% | 29.40% | 43.16% | 42.51% | 40.78% | 35.93% | 38.57% |

Reference

1. Safran M, Dalah I, Alexander J, Rosen N, Stein TI, Shmoish M, Nativ N, Bahir I, Doniger T, Krug H: **GeneCards Version 3: the human gene integrator**. *Database* 2010, **2010**:baq020.

2. Barendse W, Bunch R, Thomas M, Armitage S, Baud S, Donaldson N: **The TG5 thyroglobulin gene test for a marbling quantitative trait loci evaluated in feedlot cattle**. *Animal Production Science* 2004, **44**(7):669-674.

3. Burrell D, Moser G, Hetzel J, Mizoguchi Y, Hirano T, Sugimoto Y, Mengersen K: **Meta analysis confirms associations of the TG5 thyroglobulin polymorphism with marbling in beef cattle**. In: *29th International conference on animal genetics ISAG, Tokyo: 2004*.

4. Fortes MR, Curi RA, Chardulo LAL, Silveira AC, Assumpção ME, Visintin JA, Oliveira HNd: **Bovine gene polymorphisms related to fat deposition and meat tenderness**. *Genetics and molecular biology* 2009, **32**(1):75-82.

5. Smith T, Thomas M, Bidner T, Paschal J, Franke D: **Single nucleotide polymorphisms in Brahman steers and their association with carcass and tenderness traits**. *Gen Mol Res* 2009, **8**:39-46.

6. Chmurzyńska A: **The multigene family of fatty acid-binding proteins (FABPs): function, structure and polymorphism**. *Journal of applied genetics* 2006, **47**(1):39-48.

7. Atshaves BP, McIntosh AM, Lyuksyutova OI, Zipfel W, Webb WW, Schroeder F: **Liver fatty acid-binding protein gene ablation inhibits branched-chain fatty acid metabolism in cultured primary hepatocytes**. *Journal of Biological Chemistry* 2004, **279**(30):30954-30965.

8. JIANG Y-Z, LI X-W, YANG G-X: **Sequence Characterization, Tissue-specific Expression and Polymorphism of the Porcine (< i> Sus scrofa</i>) Liver-type Fatty Acid Binding Protein Gene**. *Acta Genetica Sinica* 2006, **33**(7):598-606.

9. Wang Y, Shu D, Li L, Qu H, Yang C, Zhu Q: **Identification of single nucleotide polymorphism of H-FABP gene and its association with fatness traits in chickens**. *ASIAN AUSTRALASIAN JOURNAL OF ANIMAL SCIENCES* 2007, **20**(12):1812.

10. Sasaki S, Yamada T, Sukegawa S, Miyake T, Fujita T, Morita M, Ohta T, Takahagi Y, Murakami H, Morimatsu F: **Association of a single nucleotide polymorphism in akirin 2 gene with marbling in Japanese Black beef cattle**. *BMC research notes* 2009, **2**(1):131.

11. Luo W, Cheng D, Chen S, Wang L, Li Y, Ma X, Song X, Liu X, Li W, Liang J: **Genome-wide association analysis of meat quality traits in a porcine Large White× Minzhu intercross population**. *International journal of biological sciences* 2012, **8**(4):580.

12. Cánovas A, Quintanilla R, Amills M, Pena RN: **Muscle transcriptomic profiles in pigs with divergent phenotypes for fatness traits**. *BMC genomics* 2010, **11**(1):372.

13. Chen S, An J, Lian L, Qu L, Zheng J, Xu G, Yang N: **Polymorphisms in AKT3, FIGF, PRKAG3, and TGF-β genes are associated with myofiber characteristics in chickens**. *Poultry science* 2013, **92**(2):325-330.

14. Ramos AM, Duijvesteijn N, Knol EF, Merks JW, Bovenhuis H, Crooijmans RP, Groenen MA, Harlizius B: **The distal end of porcine chromosome 6p is involved in the regulation of skatole levels in boars**. *BMC genetics* 2011, **12**(1):35.

15. Edea Z, Kim K-S: **A whole genomic scan to detect selection signatures between Berkshire and Korean native pig breeds**. *Journal of Animal Science and Technology* 2014, **56**(1):23.

16. Herault F, Vincent A, Dameron O, Le Roy P, Cherel P, Damon M: **The longissimus and semimembranosus muscles display marked differences in their gene expression profiles in pig**. 2014.

17. Saltiel AR, Kahn CR: **Insulin signalling and the regulation of glucose and lipid metabolism**. *Nature* 2001, **414**(6865):799-806.

18. Balla A, Tuymetova G, Tsiomenko A, Várnai P, Balla T: **A plasma membrane pool of phosphatidylinositol 4-phosphate is generated by phosphatidylinositol 4-kinase type-III alpha: studies with the PH domains of the oxysterol binding protein and FAPP1**. *Molecular biology of the cell* 2005, **16**(3):1282-1295.

19. Taverna E, Saba E, Rowe J, Francolini M, Clementi F, Rosa P: **Role of lipid microdomains in P/Q-type calcium channel (Cav2. 1) clustering and function in presynaptic membranes**. *Journal of Biological Chemistry* 2004, **279**(7):5127-5134.
